# Exploring the functional and immunological diversity of the fatty acid-binding protein (FABP) family in *Fasciola hepatica*

**DOI:** 10.1038/s41598-026-53684-0

**Published:** 2026-05-21

**Authors:** Alicja Kalinowska, Mateusz Pękacz, Katarzyna Basałaj, Alicja Laskowska, Agnieszka Wesołowska, Daniel Młocicki, Bruno Guigas, Anna Zawistowska-Deniziak

**Affiliations:** 1 Museum and Institute of Zoology PAS, Twarda 51/55, 00-818 Warsaw, Poland; 2 Pathogen Immunobiology Group, Institute of Experimental Zoology, Faculty of Biology, University of Warsaw, Warsaw, Poland; 3 Genomics and Transcriptomics Laboratory, Faculty of Biology, University of Warsaw, Warsaw, Poland; 4 Department of General Biology and Parasitology, Medical University of Warsaw, Warsaw, Poland; 5 Leiden University Center for Infectious Diseases, Subdepartment Research, Systemic Immunometabolism Group, Leiden University Medical Center, Leiden, The Netherlands

**Keywords:** Fatty acid-binding protein, *Fasciola hepatica*, Gene expression, Functional characterization, Immunological characterization, Diseases, Immunology, Microbiology

## Abstract

The liver flukes *Fasciola hepatica* and *Fasciola gigantica* cause fasciolosis, a zoonotic disease of major health and livestock importance. To evade host immunity, these parasites secrete immunomodulatory molecules, including fatty acid-binding proteins (FABPs), which are essential for parasite survival by mediating fatty acid uptake and transport, and by exerting anti-inflammatory effects that promote immune evasion and chronic infection. This study investigates the roles of *F. hepatica* FABPs’ in nutrient acquisition, immune modulation, and parasite survival, assessing their potential as vaccine, diagnostic, and therapeutic targets. We cloned all seven FhFABP isoforms and expressed six recombinantly in yeast. Gene expression analysis showed stage-specific patterns, with the highest levels in adult flukes. Isoform-specific analysis further highlighted stage-dependent expression dynamics, and functional characterization revealed distinct fatty acid-binding properties, suggesting adaptive roles in parasite survival. We assessed IgG responses in infected sheep, finding peak titer against FhFABP5 at 6–8 weeks post-infection (WPI) and a strong, prolonged response to FhFABP4, lasting 6–12 weeks. These results highlight FhFABPs as immunogenic targets for diagnostics or vaccines. Additionally, FhFABPs differentially modulated the activity of human monocyte-derived dendritic cells. Our findings emphasize the multifaceted roles of FhFABPs in nutrient acquisition and immune modulation, underscoring their potential as targets for vaccines, diagnostics, and anti-inflammatory therapies.

**Supplementary Information:**

The online version contains supplementary material available at 10.1038/s41598-026-53684-0.

## Introduction

Fasciolosis is a major zoonotic disease that significantly impacts the livestock sector, particularly affecting sheep and cattle productivity. This disease, caused by the liver flukes *Fasciola hepatica* and *Fasciola gigantica*, is among the most detrimental trematode infections globally^1^. Definitive hosts are primarily ruminants; however, humans and other grazing animals may also become infected through accidental ingestion of encysted metacercariae. After excystation in the small intestine, newly excysted juveniles (NEJs) penetrate the duodenal wall and migrate through the liver parenchyma to the biliary ducts. There, they mature into adult flukes and produce eggs that are excreted in bile and subsequently passed in the feces. In freshwater, eggs embryonate and release miracidia, which infect snail intermediate hosts. Within the snails, they develop into cercariae, which are released into the environment and encyst as metacercariae on aquatic vegetation^2^. Annual losses in animal productivity due to liver fluke infections are estimated to exceed US$3 billion^3^. Beyond its agricultural impact, fasciolosis is a significant zoonotic disease and a neglected tropical disease (NTD), estimated to affect 2.4 million people in more than 70 countries, with approximately 180 million people living in endemic areas at risk of infection^4^. Controlling fasciolosis remains challenging due to inaccurate diagnosis, drug resistance, and the absence of preventive measures such as vaccines. These challenges emphasize the critical need for innovative diagnostics and novel immunotherapeutic strategies.

Fatty acid-binding proteins (FABPs) are cytosolic proteins and represent a major component of the excretory-secretory products (ESP), extracellular vesicles of *F. hepatica*^5,6^, and soluble tegumental proteome of adult liver flukes^7^. FABPs are typically small proteins, approximately 15 kDa, with a conserved structure consisting of ten β-sheets and two α-helices^8^. They play a crucial role in fatty acid metabolism and transport from the host, which is vital for helminth survival as these parasites are unable to synthesize fatty acids *de novo*^9,10^.

In addition to their role in fatty acid metabolism, *F. hepatica* FABPs (FhFABPs) have been shown to bind and sequester antiparasitic drugs, thereby contributing to drug resistance^11,12^. For example, increased expression of FhFABP1 has been reported in triclabendazole (TCBZ)-resistant liver flukes, facilitating drug sequestration and resistance^13^. As TCBZ resistance is widespread^14^, there is an urgent need for new treatments and vaccine candidates. FABPs hold potential in this regard, having demonstrated immunoprotective properties in parasitic diseases such as schistosomiasis^15–17^. A vaccine incorporating the *Schistosoma mansoni* fatty acid-binding protein (Sm14) is currently in clinical trials and has shown promising results against both *F. hepatica* and *S. mansoni*^18^. In addition to their involvement in parasitic infections, FABPs have shown diagnostic potential as valuable molecular markers^19,20^. Furthermore, their immunomodulatory and anti-inflammatory properties suggest that they could be explored as potential therapeutic agents for treating autoimmune diseases and allergies^21–24^. Beyond *F. hepatica*, FABPs from other parasitic flatworms have been investigated for their roles in host-parasite interactions and immunoregulation. For example, Sm14 can induce IL-10 production by B cells both *in vitro* and *in vivo*, highlighting its immunomodulatory potential^25^. EmFABP1 from *Echinococcus multilocularis* has been reported to inhibit phagocytosis, induce apoptosis in murine macrophages, and modulate cytokine expression^26^. It has also been hypothesized that EgFABP1 from *Echinococcus granulosus* may be released from the cyst to interact with host cells, facilitating lipid exchange and potentially contributing to immunomodulatory processes^27^.

Seven different FABP isoforms have been identified in *F. hepatica*^28^, though research has predominantly focused on FhFABP1 or the native Fh12 protein, which, in fact is not a single protein but a blend of several isoforms^29^. As a result, the specific functions of individual FhFABPs remain largely unknown. Understanding these roles is critical, as it could provide insights into their contributions to pathogenesis, immune evasion, and potential as diagnostic markers or therapeutic targets.

This study aimed to explore the function of fatty acid-binding proteins in *F.* *hepatica*, focusing on their roles in parasite development, host-parasite interactions, and potential applications in disease control strategies. To this end, we cloned and expressed six yeast-derived recombinant FhFABP isoforms, examined their transcript levels across various developmental stages, characterized their binding affinities, and monitored IgG antibody responses against each isoform over the course of experimental infection in sheep. Although FhFABP1, FhFABP2, and FhFABP3 share conserved tertiary structures and up to 70% amino acid similarity, our results reveal significant differences in gene expression, ligand binding, and diagnostic potential among these isoforms. These findings highlight the distinct characteristics and functions of individual FhFABPs, while also acknowledging limitations such as the exclusion of certain isoforms from *in vitro* experiments. This work provides a foundation for future studies aimed at elucidating the in vivo roles of FhFABPs and assessing their potential in vaccine or diagnostic development.

Building on our previous findings^20^, we now demonstrate that FhFABP1, compared to other isoforms, uniquely reduces the inflammatory reaction of monocyte-derived dendritic cells (moDCs) upon lipopolysaccharide (LPS) stimulation and induces thrombospondin-1 (TSP-1) production. This distinct capability underscores its pivotal role in immune modulation and highlights its potential therapeutic relevance in mitigating inflammation-driven pathologies.

## Results

### Cloning and bioinformatic analysis of *F. hepatica* FABP isoforms

The molecular cloning of the *fabp* genes from *F. hepatica* successfully yielded distinct cDNA fragments corresponding to different *fabp* isoforms. The amplified products were identified as 396 bp for *fhfabp2*, *fhfabp3*, and *fhfabp4*, 402 bp for *fhfabp5*, 483 bp for *fhfabp6*, and 498 bp for *fhfabp7*.

**Fig. 1:**
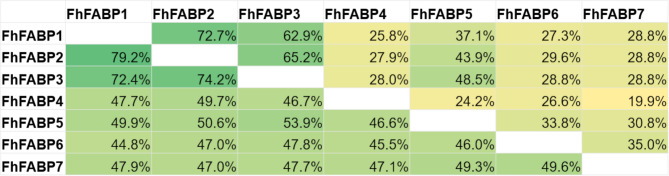
Percentage sequence identity of *F. hepatica* FABP isoforms. The values in the upper right represent the amino acid sequence identity, while the values in the lower left indicate the nucleotide sequence identity.

Sequence alignment using Clustal Omega revealed varying degrees of nucleotide identity among the *fhfabp* isoforms, ranging from 45 to 79%. This diversity was also observed at the protein level, with amino acid identities ranging from 19 to 72%. Notably, FhFABP1, FhFABP2, and FhFABP3 exhibited the highest sequence similarity, while FhFABP4 showed the least resemblance to other isoforms (Fig. 1). SignalP analysis confirmed the absence of signal peptides in all FhFABP isoforms. Using the Expasy Compute pI/Mw tool, the theoretical molecular weights for FhFABP1 through FhFABP5 were estimated to be approximately 15 kDa, while FhFABP6 and FhFABP7 were slightly larger at around 18 kDa. The predicted isoelectric points (pIs) for each FhFABP isoform are presented in Supplementary Table S5.

**Fig. 2:**
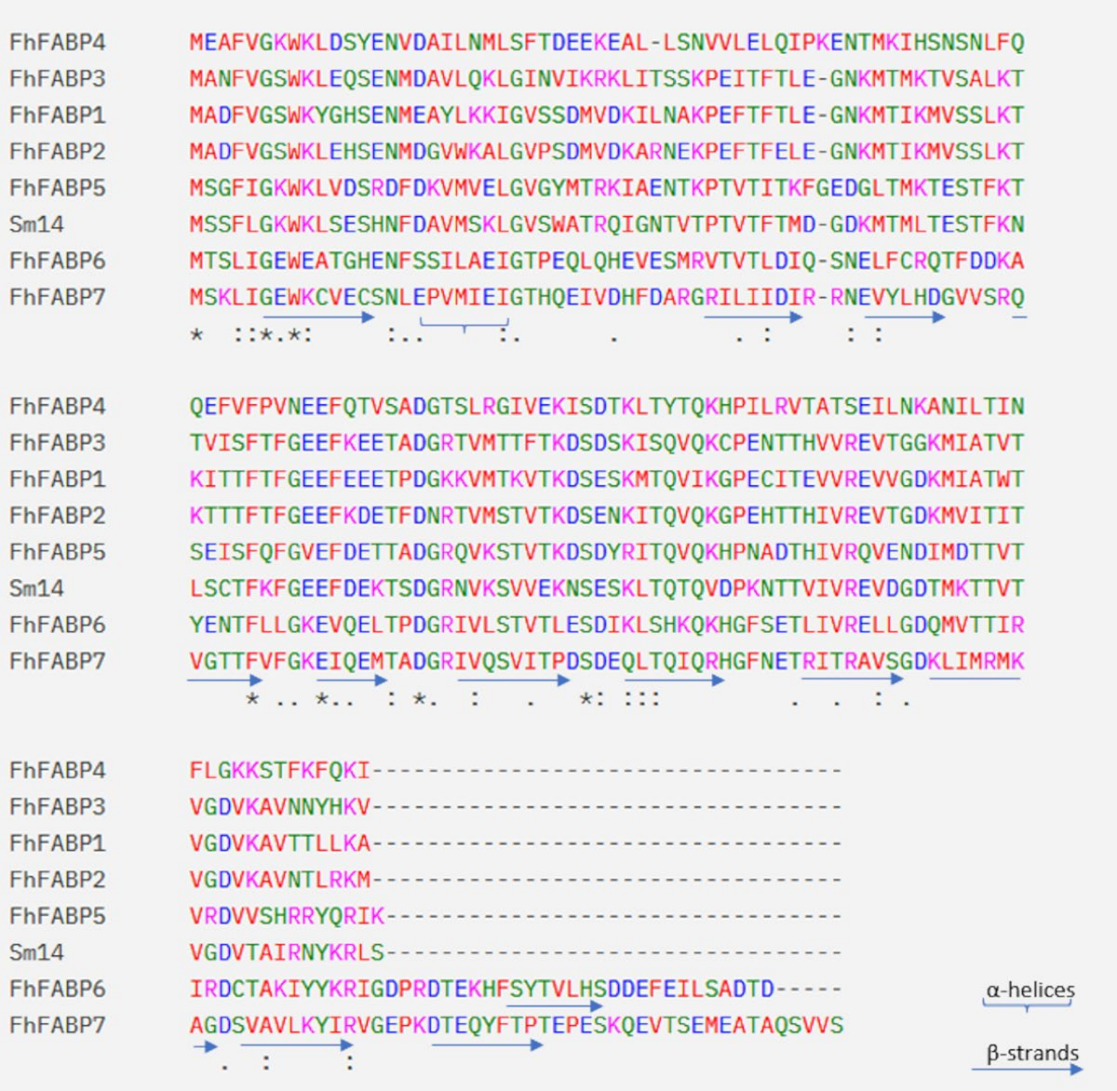
Multiple alignment of *F. hepatica* FABP isoform and Sm14 amino acid sequences. Asterisks (*) denote conserved amino acid positions, colons (:) indicate conservation among residues with strongly similar physicochemical properties, and periods (.) represent conservation among residues with weakly similar properties across *F. hepatica* FABP isoforms and Sm14. Arrows indicate the approximate positions of β-strands, while brackets denote α-helices. Secondary structure predictions were generated using the PSIPRED tool.

Secondary structure predictions using the PSIPRED tool confirmed the typical fatty acid-binding protein architecture across all FhFABP isoforms, characterized by a tandem α-helix structure and 10 β-sheets. Interestingly, FhFABP6 and FhFABP7 were found to possess an additional β-sheet at the C-terminal region. Multiple sequence alignment (Clustal Omega) identified six conserved amino acid residues (Gly6, Trp8, Phe64, Glu69, Asp76, Ser88) shared across the *F. hepatica* FABP family, suggesting strong structural and functional conservation within this protein group (Fig. 2).

### Expression of recombinant *F. hepatica* FABP isoforms

Recombinant expression of six out of seven FhFABP isoforms in *Pichia pastoris* X33 was successfully induced using methanol, resulting in detectable recombinant protein production of six FhFABP isoforms, yielding ~ 0.5–4 mg protein per liter of culture, depending on the isoform, except FhFABP2, which showed no expression*.* Additionally, FhFABP4 exhibited improved yields in minimal BMMH medium supplemented with histidine compared to the complex BMMY medium containing yeast extract and peptone, which was used for the other isoforms. Protein purification revealed distinct electrophoretic profiles for the FhFABP isoforms. FhFABP1, FhFABP3, FhFABP4, and FhFABP5 exhibited double bands around 15 kDa, suggesting potential isoform variants, post-translational modifications, or heterogeneity resulting from incomplete cleavage of the signal peptide. In contrast, FhFABP6 presented as a single band at approximately 18 kDa. Notably, FhFABP6 displayed instability, with rapid degradation observed after freeze–thaw cycles. FhFABP7 exhibited a more complex electrophoretic pattern, with molecular weights ranging from 12 to 35 kDa, and a predominant band around 24 kDa, potentially indicating extensive post-translational modification or protein aggregation (Supplementary Fig. S1).

Despite correct cloning, as confirmed by PCR screening of *Pichia* clones and sequencing, we were unable to achieve detectable recombinant expression of FhFABP2, even after multiple attempts. This isoform may have structural features or expression constraints that limit production in yeast under the conditions used, and it was therefore excluded from further analyses. These results highlight the differential expression and stability profiles of each recombinant FhFABP isoform-specific optimization even within eukaryotic hosts.

### Cross-reactivity of FhFABP1 immunized rat serum with recombinant FhFABP isoforms

Western blot analysis was performed to assess the cross-reactivity of serum antibodies from rats immunized with FhFABP1 with other recombinant FhFABP isoforms. The anti-FhFABP1 serum demonstrated broad cross-reactivity, recognizing multiple FhFABP isoforms in addition to the immunizing antigen, FhFABP1 (Supplementary Fig. S2).

Notably, FhFABP6 was not detected by the anti-FhFABP1 serum, likely due to the instability and rapid degradation of the recombinant FhFABP6 protein, which may have compromised its immunoreactivity. Interestingly, the antibodies from rat serum also cross-reacted with the recombinant fatty acid-binding protein Sm14 from *S. mansoni* (Supplementary Fig. S3). In contrast, serum from control rats (not immunized with FhFABP1) showed no reactivity toward any of the recombinant FhFABP isoforms, indicating the specificity of the immune response elicited by the FhFABP1 immunization. These findings highlight both the potential for cross-reactive antibody responses within the FhFABP family and the challenges associated with recombinant protein stability in immunological assays.

To investigate the presence of native FhFABPs in adult *F. hepatica*, polyclonal rat anti-FhFABP1 serum was used for immunodetection in both adult worm homogenates and ESP. In worm homogenates, two prominent bands were observed in immunoblots. The lower band, just below 15 kDa, corresponds to a previously characterized native FhFABP, nFh12, isolated from adult worm extracts. The higher band, below the 25 kDa marker, may represent a dimeric form of FABP or multiple isoforms within the homogenate. In the ESP, a single band of approximately 15 kDa was detected, consistent with the expected molecular weight of FhFABP isoforms (Supplementary Fig. S4). This finding suggests that adult *F. hepatica* secretes or releases FABP isoforms into its surrounding environment, despite the absence of a signal peptide, likely through a signal peptide-independent mechanism. These results highlight the potential role of FABPs in parasite-host interactions and contribute to our understanding of the composition of *F. hepatica* ESP.

### Quantitative real-time PCR analysis of *fhfabp* gene expression across *F. hepatica* developmental stages

Quantitative Real-Time PCR (qPCR) analysis revealed distinct patterns of *fhfabp* gene expression across different developmental stages of *F. hepatica*, with the highest expression observed in adult flukes. Compared to NEJ, adult flukes exhibited a 1.58-fold increase in *fhfabp* expression. The difference was even more pronounced when comparing adults to metacercariae and eggs, with a 103-fold and 813-fold increase in expression, respectively.

**Fig. 3:**
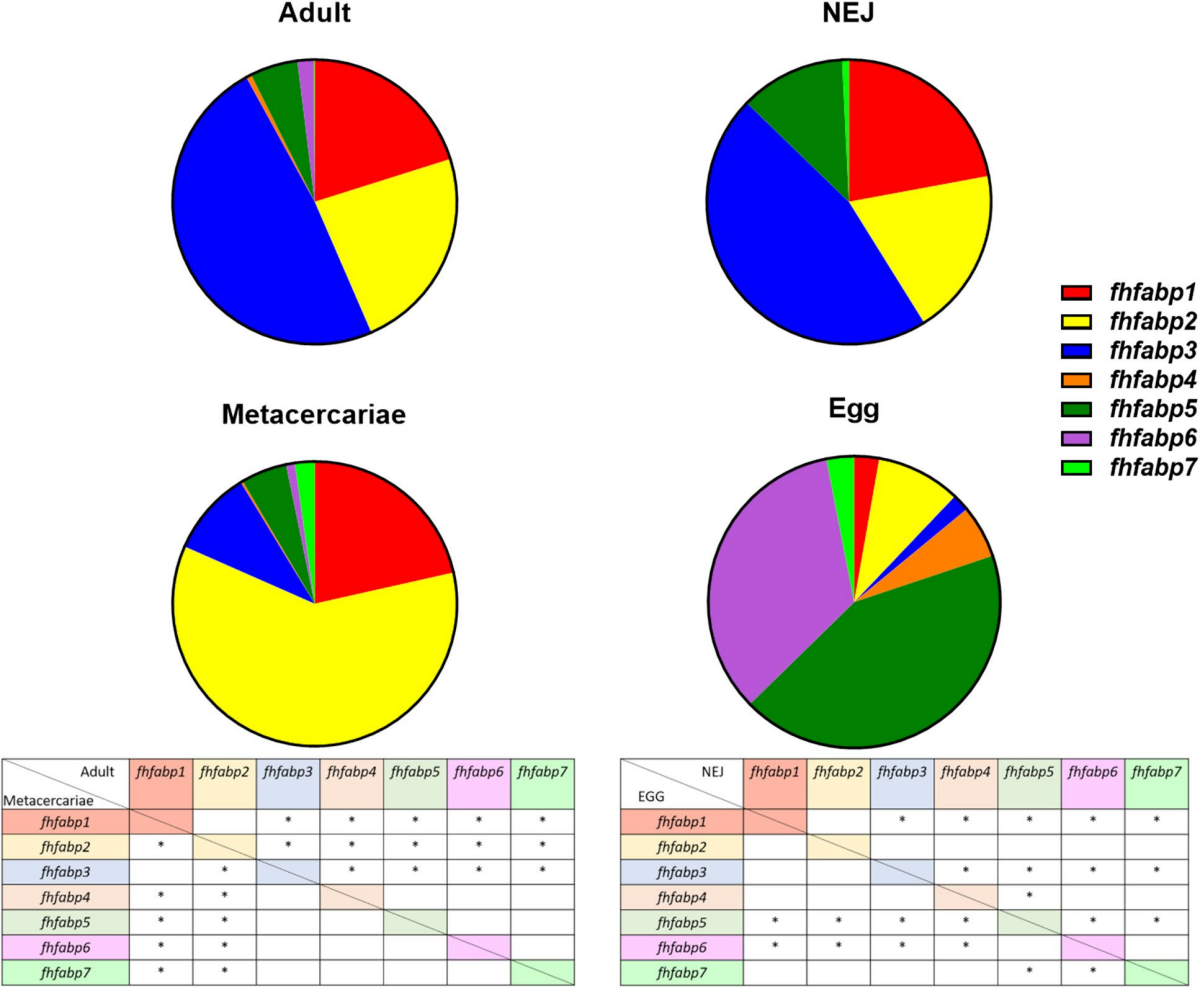
Percentage distribution of individual *F. hepatica fabp* isoforms based on mRNA expression within each developmental stage. Each circle represents 100% of the total fabp mRNA detected in that stage. Statistically significant differences between the examined *fabps* across the various developmental stages are marked with asterisks (*p < 0.05), as indicated in the accompanying table.

**Fig. 4:**
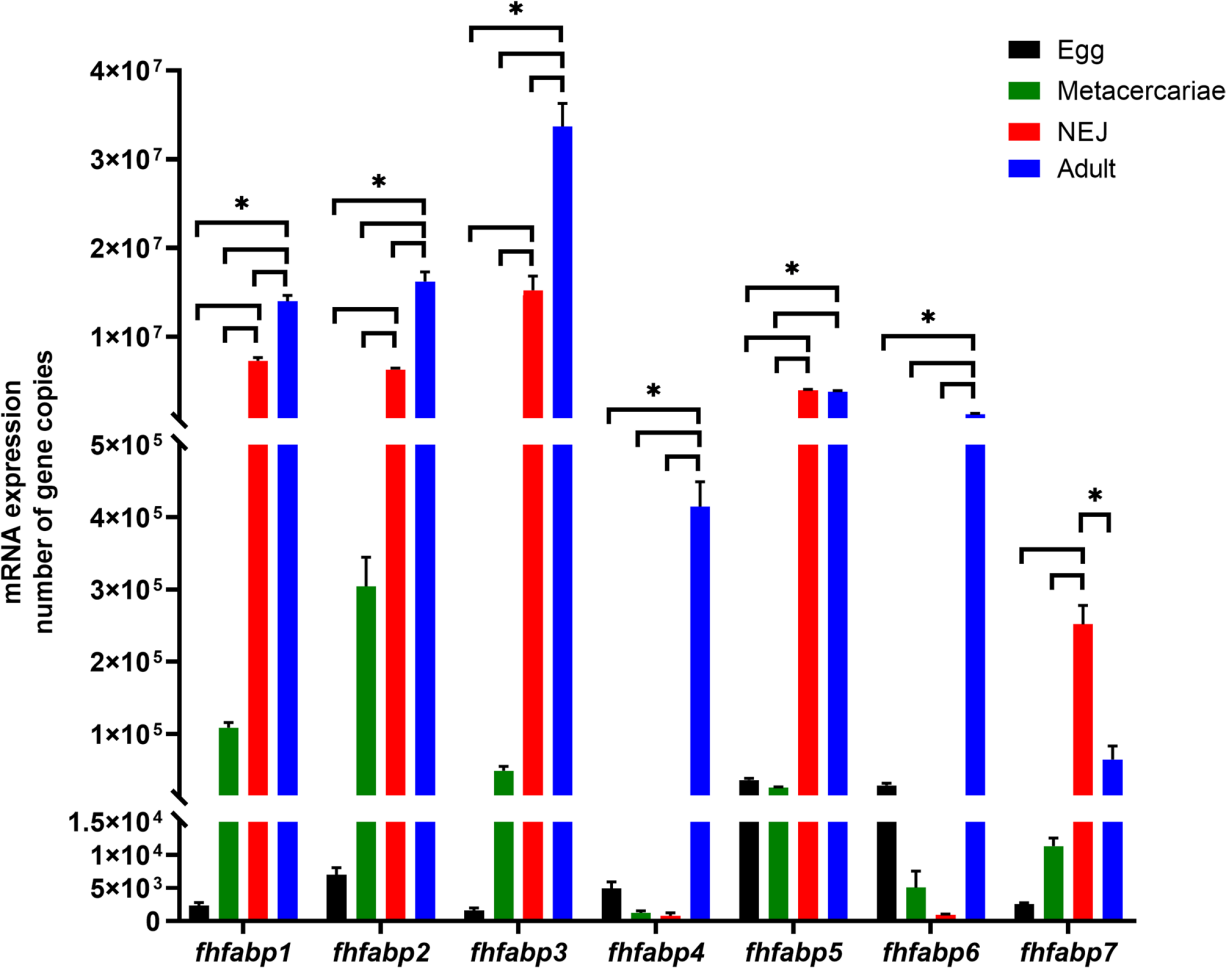
Analysis of mRNA expression level differences in *F. hepatica fabp* isoforms across various developmental stages. Results are expressed as the number of gene copies per 1 μg of total RNA and are presented as means ± SD, n = 3. Statistically significant differences between the examined groups are indicated by brackets with asterisks (*p < 0.05).

Further analysis of individual *fhfabp* isoforms showed differential expression across developmental stages. Among the isoforms, *fhfabp3* was the most abundantly expressed in both adults and NEJs, while *fhfabp2* was predominant in metacercariae. Together, *fhfabp1*, *fhfabp2*, and *fhfabp3* accounted for more than 75% of the total *fhfabp* expression in adult, NEJ, and metacercariae stages. In contrast, the egg stage showed higher expression of *fhfabp5* and *fhfabp6*, isoforms that were less prominent in later developmental stages (Figs. 3 and 4).

These findings suggest dynamic regulation of *fhfabp* isoforms throughout *F. hepatica* life cycle, indicating their potential roles in adaptation to different host environments.

### IgG antibody responses to FhFABP antigens

**Fig. 5:**
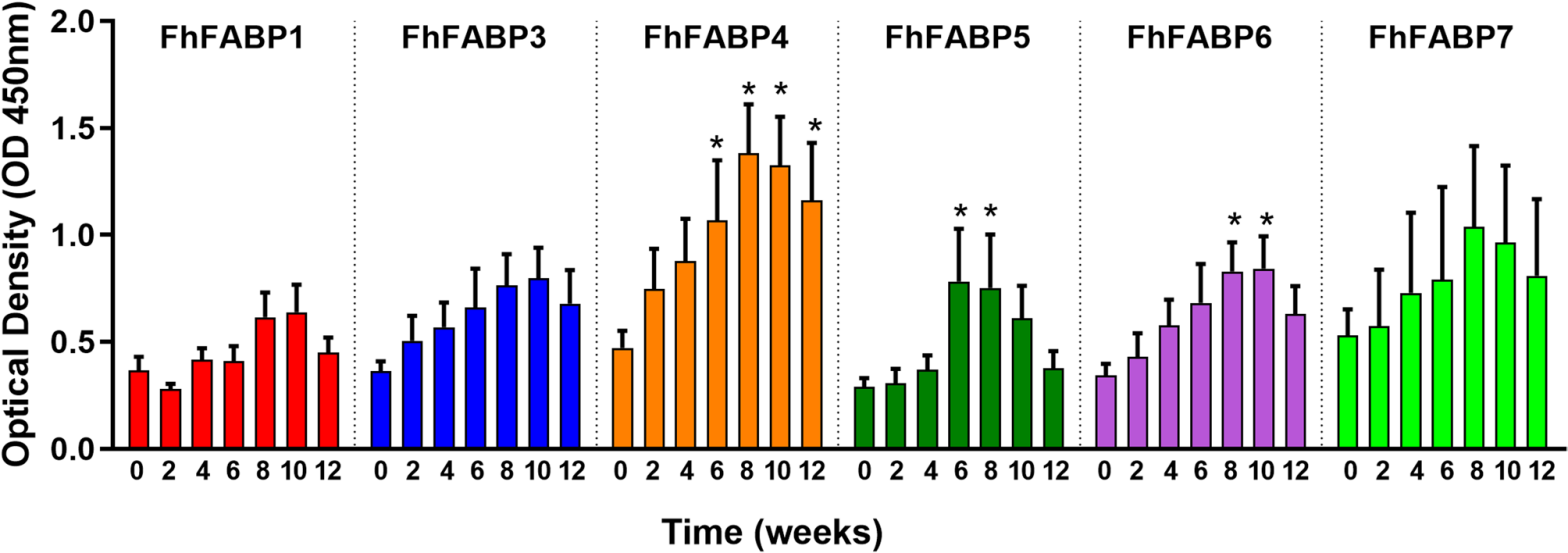
Detection of sheep IgG antibodies against recombinant *F. hepatica* FABPs. Sheep sera, collected before challenge with *F. hepatica* metacercariae and at two-week intervals up to 12 WPI, were used to probe antigens (10 μg/ml). Optical density (450 nm) results are presented as means ± SD, n = 5–8. Statistically significant differences compared to T = 0 are marked with an asterisk (*p < 0.05).

The presence of IgG specific antibodies against different FhFABP isoforms was evaluated in sheep experimentally infected with *F. hepatica*. IgG levels were assessed at seven time points post-infection (Fig. 5). IgG antibody levels against the antigens FhFABP3, FhFABP4, FhFABP6, and FhFABP7 began increasing at two WPI. In contrast, a rise in IgG levels specific for FhFABP5 was observed at six WPI, and for FhFABP1, a slight and transient increase in IgG antibody levels was detected at eight WPI. Antibody titer peaked between the 6th and 10th WPI, demonstrating a temporal pattern in the dynamics of IgG production. FhFABP4 elicited the strongest antibody response, with significantly elevated absorbance levels from the 6th WPI onwards (Fig. 5).

The antibody response to FhFABP5 peaked at weeks 6 and 8, followed by decline, while FhFABP6 induced a marked response at weeks 8 and 10 (Fig. 5). These results illustrate the variable immunogenicity of FhFABP antigens, which could have important implications for the development of diagnostic assay and vaccine based on these proteins.

### Prediction of ligand binding for *F. hepatica* FABPs

Using the COACH-D tool, ligand-binding sites and potential ligands were identified for all *F. hepatica* FABP isoforms. The probability scores reflect the confidence in the presence of ligand-binding sites, while the frequency of ligands provides insights into binding preferences. These predictions highlight both shared and isoform-specific ligand-binding patterns, indicating functional specialization among FhFABPs.

FhFABP1 exhibited the highest confidence in binding site prediction (0.91) and showed strong ligand preferences for retinol, retinal, retinoic acid, palmitic acid, and oleic acid, highlighting its affinity for both retinoids and fatty acids. FhFABP3 (0.82) displayed similar preferences, including linoleic acid and vaccenic acid. FhFABP4 had the highest confidence (0.99) and favored retinoic acid, oleic acid, and cholic acid, indicating potential sterol interactions. FhFABP5 (0.77) and FhFABP6 (0.63) displayed broader ligand preferences, with linoleic acid, palmitic acid, and cholic acid being prominent. FhFABP5 also uniquely bound troglitazone**,** an antidiabetic drug, while FhFABP6 had notable interactions with vaccenic acid. FhFABP7 (0.65) demonstrated distinct ligand preferences for retinal, bilirubin, and oleic acid, including unique ligands such as 1,3,3-trimethyl-2-[(1e,3e)-3-methylpenta-1,3-dien-1-yl]cyclohexene.

Overall, the in silico ligand predictions reveal a shared affinity for retinoids and fatty acids across all FhFABPs, emphasizing their roles in nutrient transport and host-parasite interactions. However, isoform-specific ligand preferences suggest functional diversification, with FhFABP1 and FhFABP4 specializing in retinoid and sterol interactions, while FhFABP5, FhFABP6, and FhFABP7 exhibit broader ligand versatility.

### Fatty acid binding affinity

The binding affinities of FhFABP isoforms for five fatty acids (retinoic acid, palmitic acid, myristic acid, oleic acid, and linoleic acid) were evaluated using the ANS (1-anilinonaphthalene-8-sulfonic acid) displacement assay. However, for unclear reasons, FhFABP6 and FhFABP7 did not bind to ANS and were therefore excluded from the further analysis.

**Fig. 6:**
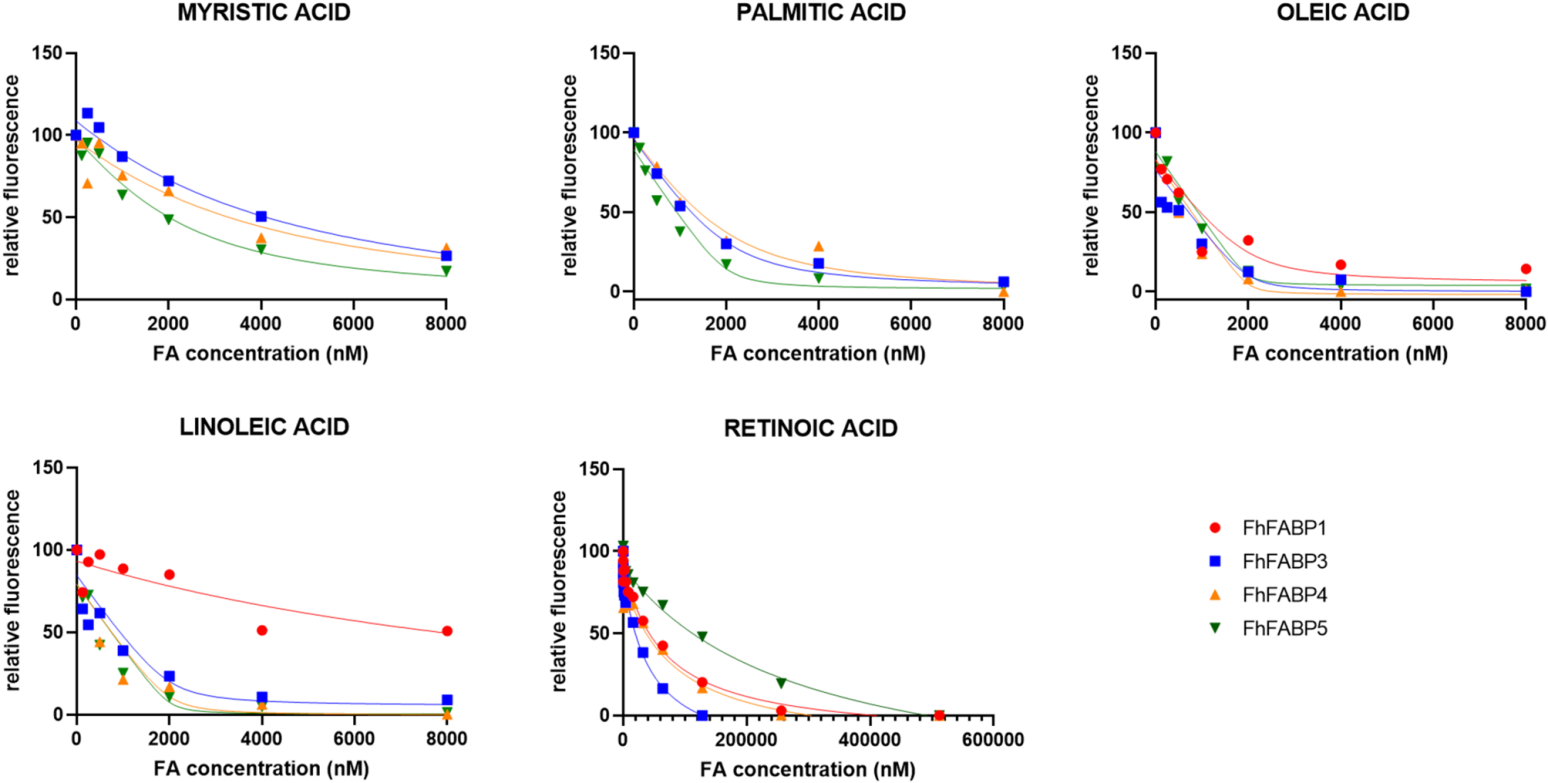
ANS displacement assay of selected fatty acids for FhFABP1, FhFABP3, FhFABP4, and FhFABP5. The binding affinity (Kd) of each FhFABP to the fatty acids was calculated based on the reduction of fluorescence. Each fitting curve represents the mean of three independent experiments, analysed using GraphPad Prism 8.0 software.

Among the remaining isoforms, retinoic acid showed the weakest binding across all tested proteins, while oleic acid exhibited the strongest affinity. FhFABP3 and FhFABP4 displayed similarly strong binding to oleic and linoleic acids and moderate binding to myristic acid, suggesting a potential role in transporting a broader range of fatty acids. In contrast, FhFABP5 bound linoleic, palmitic, and myristic acids more strongly than the other isoforms and showed the lowest affinity for retinoic acid. Meanwhile, FhFABP1 showed distinct binding behavior, with significant differences in its affinity for oleic and linoleic acids, but no binding was detected for myristic and palmitic acids (Fig. 6). These findings indicate that different FhFABP isoforms may have specialized functions in fatty acid transport and metabolism in *F. hepatica*, with some isoforms being more versatile in their fatty acid binding capabilities than others.

### Modulation of moDCs by *F. hepatica* FABPs in the presence of LPS

**Fig. 7:**
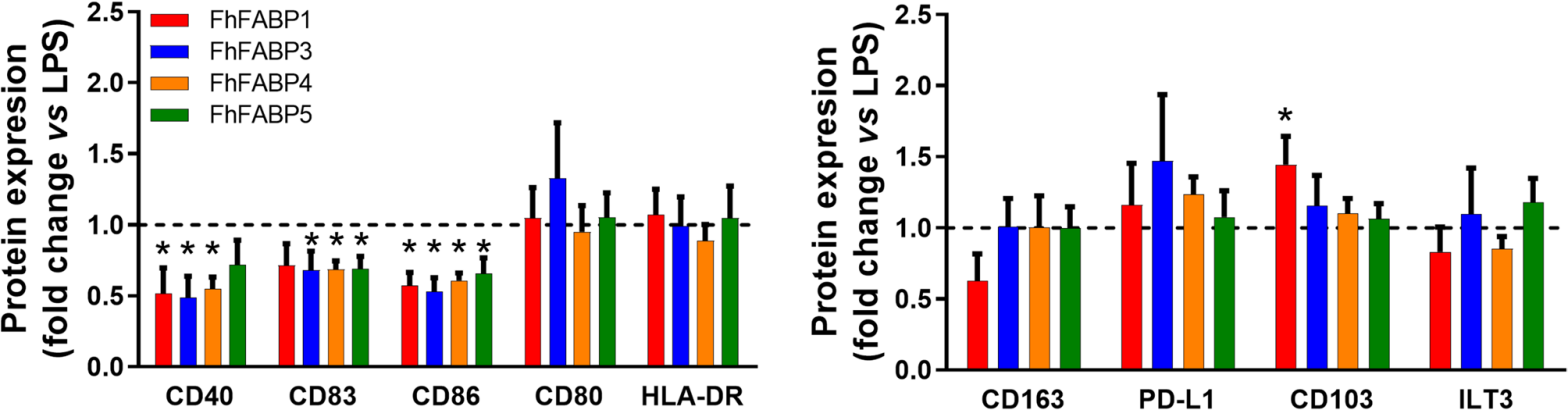
Analysis of surface marker expression on LPS-stimulated moDCs treated with *F. hepatica* FABPs. Human moDCs were treated for 48 h with 25 µg/ml recombinant *F. hepatica* FABP1, FABP3, FABP4, or FABP5 in the presence of LPS (100 ng/ml). Surface expression of activation markers (CD40, CD83, CD86, CD80, HLA-DR) and regulatory markers (CD163, PD-L1, CD103, ILT3) was measured. Data are expressed as fold change relative to moDCs treated with LPS alone (dashed line) and presented as mean ± SEM. *P ≤ 0.05 vs. LPS alone; n = 5–8 independent experiment.

**Fig. 8:**
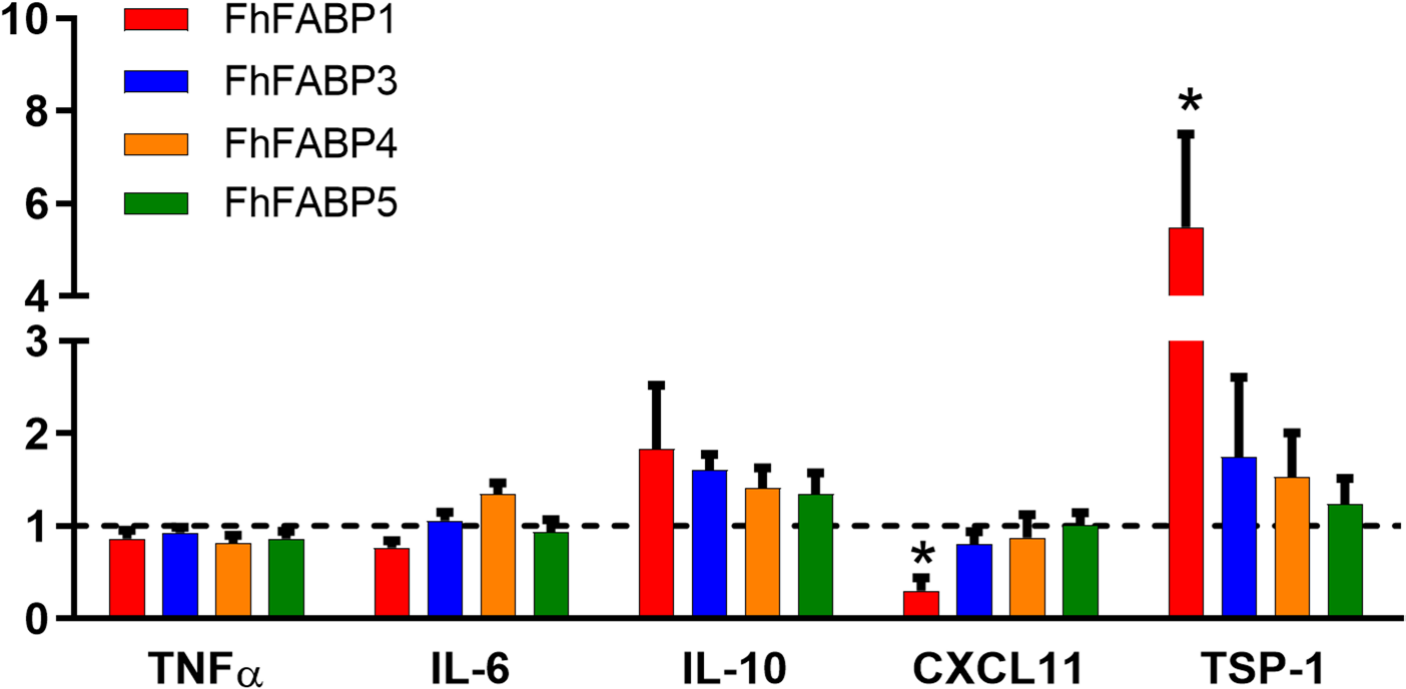
*F. hepatica* FABPs selectively modulates cytokine and chemokine production in LPS-stimulated dendritic cells. Human moDCs were treated for 48 h with 25 µg/ml recombinant *F. hepatica* FABP1, FABP3, FABP4, or FABP5 in the presence of 100 ng/ml LPS. Cytokine (TNFα, IL-6, IL-10) and chemokine (CXCL11) levels, as well as TSP-1 production, were assessed. Data are expressed as fold change relative to LPS-treated DCs alone (dashed line) and presented as mean ± SEM. *P ≤ 0.05 vs. LPS alone; n = 5–8 independent experiments.

Stimulation of moDCs with LPS in the presence of *F. hepatica* FABPs revealed significant immunomodulatory effects, with distinct outcomes for different isoforms. All isoforms were effective in reducing the expression of key maturation markers on moDCs, such as CD40, CD83, and CD86, compared to LPS stimulation alone, whereas HLA-DR and CD80 were unaffected. Notably, FhFABP1 was the only isoform to induce a significant increase in the tolerogenic marker CD103, whereas other tolerogenic markers, including PD-L1, CD163, and ILT3, were not modulated by any FhFABP isoform (Fig. 7). FhFABP1 exhibited the most pronounced effects, significantly increasing the production of TSP-1 while reducing the secretion of pro-inflammatory cytokines CXCL11 and IL-6. In contrast, TNFα levels remained relatively unchanged across all isoforms. Other FhFABPs, including FhFABP3, FhFABP4, and FhFABP5, did not significantly affect the secretion of CXCL11, or TSP-1 (Fig. 8). These findings suggest that all FhFABPs share the ability to suppress moDC maturation while highlighting the unique role of FhFABP1 in promoting a tolerogenic phenotype through enhanced TSP-1 secretion and reduced inflammatory cytokine production.

## Discussion

Fatty acids are crucial cellular components in all living organisms, serving key roles as primary constituents of biological membranes essential for cell growth and division. Additionally, they act as energy source and vital signaling molecules regulating diverse cellular processes. However, parasitic flatworms face a metabolic limitation, as they can neither synthesize fatty acids de novo nor desaturate dietary fatty acids. Consequently, they rely on host originated fatty acid, with uptake and transport being mediated by FABPs. Recent studies across multiple species, including humans, have demonstrated that despite high structural conservation and sequence similarity, FABP isoforms can exhibit distinct fatty acid binding affinities and specificities, indicating functional divergence among isoforms^30,31^. Differences in fatty acid-binding properties likely arise from subtle amino acid variations within the ligand-binding cavity that affect ligand interactions^32^. This was corroborated by our ANS displacement assay, which strongly suggested that each FhFABP isoform may play distinct roles within the organism. Interestingly, unlike the other tested FhFABPs, FhFABP1 exclusively binds to unsaturated fatty acids. This specificity is noteworthy, as unsaturated fatty acids are associated with anti-inflammatory properties^33,34^ and may activate specific signaling pathways in host immune cells, a feature that may underlie the observed ability of FhFABP1 to reduce inflammation and induce TSP-1 secretion in moDCs^22^.

Beyond their role in fatty acid metabolism, FABPs have been implicated in immune modulation and may interact with fatty acid ligands to modulate host immune responses^23,35^. Additionally, FABPs may contribute to anthelmintic resistance through drug sequestration, highlighting their potential as targets for vaccine development^10^. However, the specific functions of individual FABP isoforms remain poorly understood, and further characterization is necessary (Table 1).

**Table 1:**
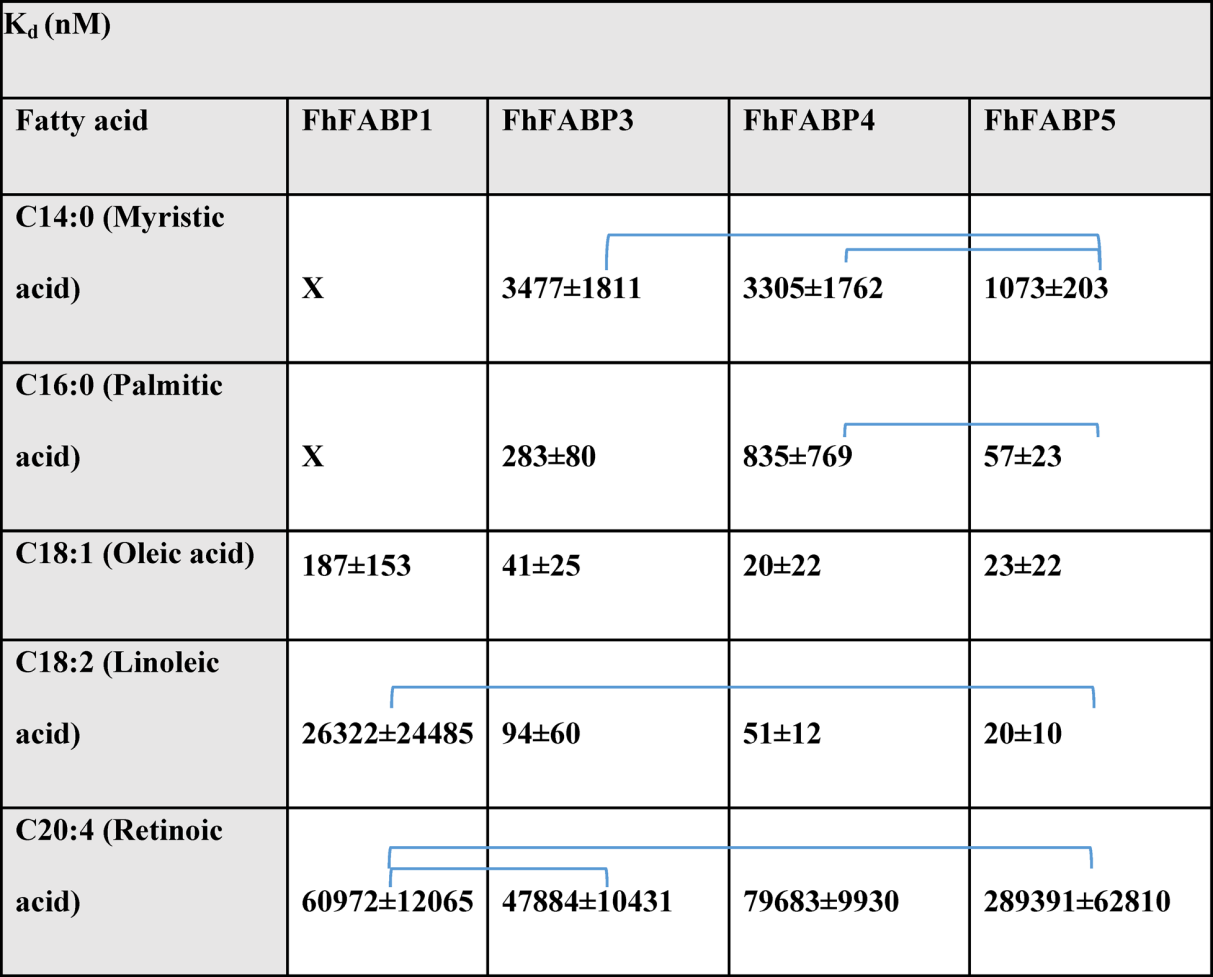
Binding affinities (Kd) of FhFABP1, FhFABP3, FhFABP4, and FhFABP5 to selected fatty acids. Kd values are presented as mean ± SD, n = 3 independent measurements. Statistically significant differences between the examined groups are indicated by brackets (*p* < 0.05).

In this study, we successfully cloned all FABP isoforms and expressed six out of seven identified from *F. hepatica* using the eucaryotic yeast (*P. pastoris*) system. This was chosen for its ability to produce high yields of properly folded recombinant proteins in a cost-effective manner. Additionally, it can secrete these proteins into the culture medium, simplifying the purification process^36^. Bioinformatic analysis of the FhFABP6 and FhFABP7 isoforms revealed longer amino acid sequences compared to typical FABPs. Their predicted secondary and tertiary structures displayed features characteristic of the FABP fold, with additional β-sheets at the C-terminus, although the confidence in these predictions was low. A similar structural feature was observed in FABP4 from *E. multilocularis,* which suggests that these elongated C-terminal regions might represent a unique variant of FABPs^37^. Functional characterization using SVMProt prediction, as performed by Morphew et al.^28^, identified FhFABP6 as a zinc-binding protein. Despite this atypical function, FhFABP6 was classified as an FABP based on its highly conserved cytosolic fatty acid-binding domain. However, the FhFABP6 protein we obtained underwent rapid degradation, and along with the FhFABP7 isoform, did not exhibit binding to ANS, which posed challenges for a more comprehensive analysis. These findings suggest that FhFABP6 and FhFABP7 may represent a subclass of FABPs with atypical function, potentially linked to their elongated sequences and uncharacterized C-terminal folds. The lack of binding to ANS further emphasizes the need to explore alternative functions or ligands for these isoforms, suggesting the potential existence of a novel FABP variant.

Interestingly, while FABPs are generally cytosolic, they have been detected in excretory-secretory products and extracellular vesicles (EVs) of *F. hepatica*^6^, suggesting a broader role in parasite-host communication. This is particularly evident for FhFABP1, which not only transports fatty acids but also modulates host immune responses by promoting TSP-1 secretion in moDCs, thereby reducing their inflammatory activity. This observation aligns with its specific binding affinity for unsaturated fatty acids, which are known to regulate immune pathways through anti-inflammatory mechanisms. Oleic acid is well-documented for its anti-inflammatory properties. One of its key mechanisms involves the modulation of the NF-κB signaling pathway. Oleic acid suppresses the activation of NF-κB, which regulates the expression of pro-inflammatory cytokines, thereby reducing inflammatory responses. Additionally, oleic acid promotes the polarization of macrophages toward the anti-inflammatory M2 phenotype. Another mechanism involves the activation of Peroxisome Proliferator-Activated Receptors (PPARs), particularly PPAR-γ, which helps to reduce the expression of inflammatory markers^33^. In contrast, FABPs like FhFABP3, FhFABP4, and FhFABP5 may bind a broader range of fatty acids, potentially leading to less targeted or distinct cellular effects. Our findings confirmed the presence of native FhFABPs in both adult homogenates and ES products, in agreement with previous studies^5,7^. However, we were unable to detect FhFABPs in other developmental stages, such as eggs, metacercariae, NEJ, and their ES products. This is likely due to the low levels of FhFABP expression in these stages, which may have been below the detection threshold of our assays, including Western blot analysis. This suggests that FhFABP expression could be stage-specific, with significantly higher expression in adults where nutrient acquisition and interaction with the host environment are more prominent. Nevertheless, our study demonstrates the mRNA expression of *fabps* across all tested life cycle stages of *F. hepatica*, with particularly high levels observed in adult and NEJ forms. These findings are consistent with previous studies on other digenean trematodes, such as *S. mansoni*^38^, *F. gigantica*^39^, and *Opisthorchis viverrini*^40^, where FABPs were also present throughout the life-cycle. This stage-specific expression pattern suggests that FABPs may be essential for parasite survival at different points in the life cycle, particularly in stages where nutrient acquisition from the host is critical.

We observed distinct expression patterns for various *fabp* isoforms, with *fhfabp3* showing the highest expression in adult and NEJ stages, while *fhfabp2* was predominant in metacercariae, and *fhfabp5* and *fhfabp6* were specifically expressed in eggs. This suggests functional specialization among isoforms, with FhFABP1 adapted to modulate host immune responses during the adult and NEJ stages, while others, like FhFABP5 or FhFABP6, may fulfill complementary roles, such as reproduction or nutrient transport. The identical sequence of FhFABP3 to its counterpart in *F. gigantica* suggests a conserved function within the *Fasciola* genus. The differential expression of multiple FABP isoforms may reflect their functional specialization in acquiring a broader range of fatty acids from the host, compensating for the parasite’s lack of fatty acid synthesis^31^.

To date, no immunolocalization studies have been conducted to map the distribution of FABPs in *F. hepatica*. However, in *F. gigantica*, FABPs have been localized in parenchymal tissue and the oral and ventral suckers of both adult and juvenile parasites^39^. Similarly, in *O. viverrini*, FABPs were found in parenchymal cells, oral sucker muscles, eggs, testes, and the tegument of adult parasites, while Sm14 in *S. mansoni* was detected near host interfaces, such as the basal lamella of the tegument and the epithelium of the gut^38,40^. These findings suggest that FABPs may facilitate the absorption and transport of fatty acids. Additionally, sex-specific localization of FABPs has been observed in several parasitic fluke species. For example, in *Schistosoma japonicum,* reactivity of anti-Sj14 antibodies was observed in vitelline droplets of females and lipid droplets beneath the subtegumental region of males^41^. In male *S. mansoni*, Sm14 was detected in the basal lamellae of the gut and tegument. Similarly, in *F. gigantica,* a reproductive organ-specific distribution was observed, with FgFABP1 localized to male reproductive tissues and FgFABP3 supporting female reproduction through localization in vitelline cells^39^. These findings highlight a significant role for FABPs in nutrient acquisition and potentially in reproductive processes and modulation of host immune responses. However, further studies on FhFABPs are required to validate these hypotheses and fully understand their functions.

Several studies have demonstrated the promising diagnostic potential of *Fasciola* derived FABPs for detecting fasciolosis in both animals and humans^19,20,29^. However, much of this research has focused on native FABPs isolated from adult flukes, which likely consist of a mixture of different isoforms, predominantly FABP1-FABP3. Espino and colleagues^29^ compared the diagnostic reactivity of native FABPs and recombinant FABP1 (formerly rFh15) and found that the native protein exhibited superior reactivity. Specifically, antibodies against native FABP (nFh12) appeared in infected rabbits as early as the second WPI, whereas antibodies against rFh15 only emerged around the sixth WPI. These findings suggest that FABP1 (rFh15) may be less immunogenic or immunoprotective than other components of the nFh12 protein complex. Similarly, our results indicated a low antibody response against FhFABP1.

In contrast, Morphew et al.^28^ reported a robust FABP5-specific IgM and IgG response between the second and fourth WPI, whereas FABP1 and FABP3 were only weakly recognized during natural infections, suggesting that FABP5 holds significant potential as a diagnostic marker for fasciolosis. In our study, we detected high antibody titer against FhFABP5 between the sixth and eighth WPI, while FhFABP4 elicited the most sustained and robust antibody response, persisting from the sixth to the twelfth week.

Our findings highlight the diverse features of FABPs and their potential roles in the biology of *F. hepatica*, particularly in nutrient acquisition and immune modulation. The ability of FhFABP1 to specifically reduce inflammation and induce TSP-1 secretion further distinguishes it as a key factor in host-parasite interactions. These results lay the groundwork for further investigation into the structural and functional differences among FhFABP isoforms, particularly FhFABP1, and their roles in facilitating parasite survival and modulating host immunity. While this study offers detailed insights into the mRNA expression patterns and ligand-binding properties of FhFABP isoforms, future work should also focus on localizing these proteins within adult fluke and NEJ tissues to better define their physiological roles. Such information would complement our findings and help clarify the functional specialization of individual isoforms.

## Materials and methods

### FhFABP cloning and recombinant protein production

Total RNA was extracted from a single adult *F. hepatica* fluke obtained from an experimentally infected sheep and used for cDNA synthesis. This cDNA was subsequently used to amplify by PCR a series of *fhfabp* isoforms, including *fhfabp2* (GenBank accession no. PQ619435), *fhfabp3* (GenBank accession no. AJ250098), *fhfabp4* (GenBank accession no. KJ713300.1), *fhfabp5* (GenBank accession no. KJ713302), *fhfabp6* (GenBank accession no. KJ713304), and *fhfabp7* (GenBank accession no. KJ713306), using isoform-specific primers. PCR amplification was performed using Phusion High-Fidelity or DreamTaq DNA Polymerase (Thermo Fisher Scientific) under standard conditions recommended by the manufacturer. Detailed information on annealing temperatures, polymerase type, extension times, and cycle numbers for each isoform is provided in Supplementary Table S1. The complete coding sequences were cloned into pPICZα expression vector (Invitrogen), utilizing the following restriction enzyme sites: *PstI* and *XbaI* for *fhfabp2*, *fhfabp3, fhfabp4*, and *fhfabp5*; *ClaI* and *XbaI* for *fhfabp6*; and *PstI* and *NotI* for *fhfabp7*. The *fhfabp*2 coding sequence has been submitted to the GenBank database under accession number PQ619435.

Since the *P. pastoris* expression system produces hypermannose N-glycans, potentially leading to additional glycosylation sites and bulky mannose-rich structures, we modified potential N-glycosylation sites to prevent hyperglycosylation, as previously done for other proteins such as Sm14, where this strategy effectively prevented hyperglycosylation^25^. Using the NetNGlyc 1.0 software tool (https://services.healthtech.dtu.dk/services/NetNGlyc-1.0/), we identified these sites in FhFABP2, FhFABP6, and FhFABP7. To eliminate these sites, asparagine (Asn) residues were mutated to glutamine (Gln) at specific positions: Asn77 in FhFABP2, Asn15 in FhFABP6, and Asn101 in FhFABP7. This conservative substitution is widely used, as both residues are polar and uncharged, with side chains terminating in an amide group (–CONH₂), and differ only by a single methylene group thus generally exerting minimal effects on protein structure. These modifications were introduced by inverse PCR. Primers were designed to introduce the desired point mutations. PCR was performed using Phusion High-Fidelity DNA Polymerase under standard conditions, and detailed information on primer sequences, annealing temperatures, and cycle numbers is provided in Supplementary Table S1. The PCR products were subsequently purified and phosphorylated using T4 polynucleotide kinase (PNK), followed by blunt-end self-ligation. All constructs were verified by nucleotide sequencing.

The recombinant plasmids were linearized using *PmeI* and transformed into *P. pastoris* X33 strain by electroporation. Isolated colonies were grown, and protein expression was induced in BMMY and BMMH media with 0.5% methanol at 29 °C for up to 96 h, with methanol supplementation every 24 h. The expressed proteins were purified using a nickel-nitrilotriacetic acid (Ni–NTA) affinity chromatography column (Macherey–Nagel), followed by concentration and dialysis against PBS using the AMICON ultrafiltration system (Merck). Endotoxins were removed using Endotoxin Removal Spin Columns (Pierce), and the protein solutions were filtered through a 0.22 μm syringe filter.

FhFABP1 (GenBank accession no. M95291) was produced following the method of Zawistowska-Deniziak et al.^22^. Briefly, the coding sequence was cloned into the pPICZαB vector and expressed in *P. pastoris* (X33 strain), followed by purification using Ni–NTA affinity chromatography. Purity and presence of the recombinant FhFABPs were assessed by SDS-PAGE and Western blot, using anti-polyhistidine peroxidase-conjugated antibody (Sigma). Protein concentration was determined using the Pierce BCA Protein Assay Kit (Thermo Scientific).

### ELISA analysis

Ninety-six-well microplates were coated with 10 μg/ml of each recombinant protein (FhFABP1, FhFABP3, FhFABP4, FhFABP5, FhFABP6 and FhFABP7) in carbonate coating buffer, (pH 9.6) and incubated overnight at 4 °C. After three washes with PBS containing 0.05% Tween 20 (PBST), wells were blocked with 5% skim milk in PBST for 1 h at 37 °C. Following another three washes, sera (commencing at a 1:10 dilution in 1% skim milk) from sheep experimentally infected with *F. hepatica* metacercariae (150 metacercariae administered orally) were added and incubated for 1 h at 37 °C. Blood samples were collected biweekly from these animals from pre-infection until 12 WPI, as previously described^42^. After serum incubation, wells were washed, and rabbit anti-sheep IgG (H + L chain) HRP-conjugated antibodies (diluted 1:100,000, Bethyl Laboratories) were added, followed by a 1 h incubation at 37 °C. Plates were washed five times with PBST before adding 3,3,5,5-tetramethylbenzidine (TMB) substrate solution (Sigma-Aldrich) and incubating for 20 min at room temperature (RT). The reaction was stopped with 2 M sulfuric acid, and absorbance was measured at 450 nm using a Synergy HT spectrophotometer (BioTek). Optical density (OD) values at 450 nm were recorded and values above established cut-off thresholds were reported. Cut-off thresholds were established as the mean OD of negative control samples plus three standard deviations (mean + 3 SD). Statistical analysis was performed to evaluate changes in antibody levels against individual *F. hepatica* antigens during infection. Data were analysed using two-way analysis of variance (two-way ANOVA) with “time post infection” and “antigen” as factors. As the aim of the analysis was to assess temporal changes for each antigen separately, only the effect of the “time” factor within each antigen was interpreted, without comparing antibody responses between different antigens. When significant differences were found, Fisher’s Least Significant Difference (LSD) post-hoc test was applied to compare each timepoint with the baseline (timepoint 0, before infection). The assumptions of ANOVA were verified based on residuals inspection in GraphPad Prism. Statistical analyses were performed in GraphPad Prism 8.0 (GraphPad Software, La Jolla, USA), with a significance level of α = 0.05.

### Preparation of rat anti-FhFABP1 serum and western blotting

Rat polyclonal anti-FhFABP1 serum was prepared by immunizing a Wistar rat with 100 μg of recombinant FhFABP1 protein, followed by three booster doses (75 μg, 50 μg, and 25 μg) at two week intervals. One week after the final booster, the rat was euthanized, and blood was collected. Serum was separated by centrifugation and stored at -70 °C. All animal experiments were conducted following relevant guidelines and regulations, and ethical approval was obtained from the 2nd Local Ethical Committee for Animal Experimentation of Warsaw University of Life Sciences-SGGW, Poland, under approval number WAW2/080/19. The manuscript reporting adheres to the recommendations in the ARRIVE guidelines.

Adult *F. hepatica* flukes were recovered from the livers of experimentally infected sheep. To obtain protein extracts and excretory–secretory (ESP) antigens, adult flukes were washed in PBS, preincubated in RPMI 1640 medium at 37 °C for 2 h, and then incubated for ~ 24 h in fresh RPMI 1640 supplemented with 100 U/ml penicillin and 0.1 mg/ml streptomycin. Culture media were collected every 2–3 h, replaced with fresh medium, and immediately frozen at –80 °C. Pooled media were centrifuged and concentrated AMICON ultrafiltration system (Merck) with a 3 kDa cut-off. For preparation of adult fluke protein extracts, parasites were homogenized in sterile PBS using a glass homogenizer, followed by sonication and centrifugation. Protein concentration was determined using the Pierce BCA Protein Assay Kit (Thermo Scientific).

For Western blotting, recombinant FhFABPs (2 μg each), adult *F. hepatica* protein extracts (15 μg) and ESP antigens (15 μg) were separated on 15% SDS–polyacrylamide gels and transferred to nitrocellulose membranes. The membranes were blocked with 5% skim milk in PBS for 1 h, followed by overnight incubation at 4 °C with rat anti-FhFABP1 serum or non-immunized rat serum (diluted 1:1000). After three PBST washes, membranes were incubated with goat anti-rat IgG HRP-conjugated antibody (1:10,000 dilution) for 2 h at room temperature. Protein bands were visualized using the SuperSignal™ West Pico PLUS Chemiluminescent Substrate (Thermo Scientific).

### RNA extraction and cDNA synthesis

Total RNA was extracted from various life stages of *F. hepatica*, including eggs, metacercariae, NEJ, and adult flukes. Metacercariae were obtained from cercariae shed by infected snails, and NEJ were excysted in vitro. Adult flukes and eggs were collected from experimentally infected sheep. All parasite stages were derived from our laboratory-maintained *F. hepatica* strain (“CVL Weybridge”), originally obtained from the UK and continuously propagated since 2003^43^. RNA extraction was performed using Trizol reagent (A&A Biotechnology). Prior to the addition of the Trizol solution, the parasite stages were homogenised either with a mortar and pestle in the presence of liquid nitrogen (eggs and metacercariae) or with a tissue grinder (NEJ and adult flukes). The extraction of RNA from these samples was performed utilizing the Total RNA Isolation Kit (A&A Biotechnology), adhering strictly to the protocol provided by the manufacturer. RNA was eluted in 100 µL of RNase-free water. Purified RNA was treated with DNase to remove genomic DNA contamination, and RNA concentration and purity were determined using a NanoDrop spectrophotometer. Equal amounts of total RNA were used for cDNA synthesis with RevertAid™ H Minus M-MuLV Reverse Transcriptase (MBI Fermentas).

### Quantitative real-time PCR analysis for *fhfabp* isoform expression

Transcript levels of *fhfabp* isoforms were quantified in triplicate using the QuantStudio 6 Real-Time PCR system (Applied Biosystems™), in accordance with the manufacturer’s fast cycling protocol employing PowerUp™ SYBR™ Green Master Mix (Applied Biosystems™). The qPCR conditions included Uracil-DNA Glycosylase (UDG) activation, initial denaturation, and 40 amplification cycles with denaturation at 95 °C and annealing/extension at 60 °C. Reactions were performed in 10 µl volumes using 1 µl of cDNA, SYBR™ Green Master Mix, 0.6 µM primers, and nuclease-free water. Primer pairs specific to each *fhfabp* isoform were designed and validated for selectivity to ensure no cross-priming occurred with non-target isoforms, as described in Supplementary Tables S2 and S3. Specificity was first evaluated in silico and then confirmed experimentally using all seven plasmids encoding the respective *fhfabp* isoforms, with no evidence of substantial cross-reactivity among *fhfabp* isoforms. A standard curve for absolute quantification was generated by serial dilutions of a plasmid containing the target *fhfabp* isoform sequence, ranging from 1 × 10^6^ down to 10 copies per reaction*.* The copy number of *fabp* per microgram of total RNA from each developmental stage of the parasite was calculated based on the Cq values obtained during the annealing/extension phase of the qPCR.

Statistical analyses to discern significant expression differences across the various life cycle stages of *F. hepatica* and between each *fabp*s were conducted using the one-way ANOVA with Tukey’s post hoc test. Results are presented as means ± SD, n = 3. Differences between groups were considered statistically significant at *p < 0.05.

### Predicting potential ligands for protein binding

The COACH-D tool^44^, an advanced version of the original COACH algorithm, incorporates multiple sophisticated prediction algorithms, including COFACTOR, TM-SITE, and S-SITE, to forecast potential ligands for protein binding. This in silico tool leverages deep learning techniques and a larger, more refined database of known protein–ligand interactions, significantly boosting the reliability and accuracy of its predictions. COACH-D excels in identifying key biological binding sites by using sequence homology and structural alignment techniques, and it features improved usability and integration capabilities. This method not only predicts binding sites but also suggests the most likely ligands, providing a valuable resource for understanding protein function.

### ANS displacement assay for fatty acid binding affinity

The ANS displacement assay, utilized to evaluate the binding affinity of recombinant *F. hepatica* FABPs for various fatty acids, began with preparing a 16 mM stock solution of ANS (Cayman Chemical) in DMSO. Fatty acids, including palmitic acid, and myristic acid in sodium salt form, and oleic, linoleic, and retinoic acids in free fatty acid (FFA) form, were obtained from Sigma-Aldrich.

Stock solutions of the fatty acids were prepared at 10 mM in different solvents based on their solubility: absolute ethanol for oleic and linoleic acids, DMSO for retinoic acid, and 0.01 N sodium hydroxide (NaOH) for stearic, palmitic, and myristic acids. Myristic acid stock solution was maintained at 50 °C, while stearic and palmitic acid solutions were heated to 85 °C to ensure complete dissolution.

Following the protocol by Liu et al.^45^, recombinant FhFABPs (2 µM) were mixed with ANS (4000 nm) in a reaction buffer consisting of 10 mM potassium phosphate (pH 7.4) and 40 mM potassium chloride (KCl). The mixture was then titrated with increasing concentrations of fatty acids to displace ANS bound to the FhFABPs. After a 2-min incubation in darkness to prevent photobleaching, fluorescence intensity was measured using a Biotek Synergy HT Microplate Reader, with excitation and emission wavelengths set at 360 nm and 460 nm, respectively, to monitor changes in fluorescence indicative of fatty acid binding.

The binding affinity or the apparent dissociation constant (Kd) of *F. hepatica* FABPs to ANS was calculated by nonlinear least-squares regression one-site binding analysis using the GraphPad Prism 8.0. The Kd of FhFABPs to selected fatty acids was calculated using the following equations^46^**:**

$$\begin{aligned} F = & F_{0} - \left\{ {\left[ {1 + \left( {P_{T} + L_{T} } \right)K_{a} - \left[ {\left( {P_{T} - L_{T} } \right)^{2} K_{a} ^{2} + 2\left( {P_{T} + L_{T} } \right)K_{a} + 1} \right]^{{1/2}} } \right]/\left[ {2P_{T} K_{a} } \right]} \right\} \\ & \left( {F_{0} - F_{{\max }} } \right) \\ \end{aligned}$$

K_d_ = 1/K_a_, where F is the measured fluorescence, F_0_ is the fluorescence in the absence of ligand, P_T_ is the total protein concentration, L_T_ is the titrant ligand concentration, K_a_ is the apparent association constant for the titrant ligand, and F_max_ is the fluorescence signal after complete saturation of the FhFABP with ligand. Statistical analyses to assess significant differences in Kd values among *F. hepatica* FABP isoforms were performed using the Kruskal–Wallis test followed by Dunn’s post hoc test.

### Human moDC culture, stimulation and analysis

Human monocytes were isolated from concentrated peripheral blood (buffy coat) donated with informed consent by healthy anonymous volunteers at Regional Center for Blood Donation and Blood Treatment (Warsaw, Poland). In accordance with national regulations, the use of anonymized blood from voluntary donations does not require additional ethical approval. Following gradient centrifugation and positive selection using CD14 MACS beads (Miltenyi Biotec, Germany), as previously described^22^, monocytes were next differentiated to moDCs using human rGM-CSF (20 ng/ml, Life technologies) and rIL-4 (0.86 ng/ml, R&D Systems) for 5–6 days, with media refreshment on day 3. Immature moDC (0.5 × 10^6^/ml) were stimulated with 100 ng/ml LPS (*E. coli* O127:B8 strain, Sigma-Aldrich) on day 5–6 in the presence or absence of *F. hepatica* recombinant molecules FABP1, FABP3, FABP4 and FABP5 (25 μg/ml each). After 48 h of stimulation, cell supernatants were collected for cytokine/chemokine analyses and cells were stained with Fixable Aqua Dead Cell Stain Kit (Invitrogen/Thermo Scientific) for determination of cell surface expression of co-stimulatory molecules by spectral flow cytometry (Norther Lights, CYTEK) using various antibodies (Supplementary Table S4). Cytokine (IL-6, IL-10, TNFα) and chemokine (CXCL11, TSP-1) concentrations were determined using commercial ELISA kits (Supplementary Table S4). All data are presented as mean ± standard error of the mean (SEM). Statistical analysis was performed using GraphPad Prism 8.0 with unpaired t-test or one-way analysis of variance (ANOVA) followed by Fisher’s *post-hoc* test. Differences between groups were considered statistically significant at *P* < 0.05.

## Supplementary Information

Supplementary Information.

## Data Availability

The nucleotide sequence generated during the current study has been deposited in GenBank under accession number PQ619435. All remaining datasets generated and/or analysed during the current study are available in the Dane Badawcze UW repository, 10.58132/QSH01A.
