# Supplementary material for: Exploring the functional and immunological diversity of the fatty acid-binding protein (FABP) family in Fasciola hepatica
Source: Sci Rep. 2026 May 21;16:23175. doi: 10.1038/s41598-026-53684-0 (PMC13396458; doi:10.1038/s41598-026-53684-0)
Supplement: Supplementary file 1 — Supplementary Information. [file 41598_2026_53684_MOESM1_ESM.pdf]

Supplementary table

Table S1. Primer sequences and PCR amplification conditions for *F. hepatica* FABP isoforms

Isoform	Purpose of PCR	Primer name	Sequence (5'→3')	Annealing Temperature (°C)/polymerase	Extension time (s)	Number of cycles
fhfabp1	Amplification for cloning	F-fhFABP1-PstI	ATTCTGCAGGGATGGCTGACTTTGTGG	60.4/ DreamTaq DNA Polymerase	30	34
		R-fhFABP1-XbaI	TCTCTAGACTCGCTTTGAGCAGAGTGG			
fhfabp2	Amplification for cloning	F-fhFABP2-PstI	TTCTGCAGTCATGGCTGACTTCGTGGGTTC	67.2/ DreamTaq DNA Polymerase	30	34
		R-fhFABP2-XbaI	CCGTCTAGAGCCATTTTGCGCAAAGTGTTC			
	Site-directed mutagenesis (N77Q)	FhFABP2_mut_F	TCGACCAGAGAACGGTTATGAG	64.5/ Phusion DNA polymerase	90	35
		FhFABP2_mut_R	AAGTCTCGTCTTTGAATTCCTCGC			
fhfabp3	Amplification for cloning	F-fhFABP3-PstI	GCGCTGCAGTCATGGCCAATTTTGTGGGTTC	72/ Phusion DNA polymerase	15	35
		R-fhFABP3-XbaI	AGGTCTAGAGCTACTTTGTGGTAATTGTTCACAG			
fhfabp4	Amplification for cloning	F-fhFABP4-PstI	GCGCTGCAGTCATGGAAGCATTCGTCGG	68.1/ Phusion DNA polymerase	15	35
		R-fhFABP4-XbaI	AGGTCTAGAGCAATTTTCTGGAATTTGAAGG			
fhfabp5	Amplification for cloning	F-fhFABP5-PstI	CCGCTGCAGTCATGTCTGGATTTATCGG	60.7/ DreamTaq DNA Polymerase	30	34
		R-fhFABP5-XbaI	GGCTCTAGATCTTTGATGCGTTGGTATCTCC			
fhfabp6	Amplification for cloning	F-fhFABP6-PstI	GCAATCGATCATGACATCACTGATCGGAG	59/ DreamTaq DNA Polymerase	30	34
		R-fhFABP6-XbaI	TAATCTAGACTGTCAGTATCCGCGGATAGG			

	Site-directed mutagenesis (N15Q)	FhFABP6_mut_F	GCCACGAACAGTTCTCATCTATC	63.1/ Phusion DNA polymerase	90	35
		FhFABP6_mut_R	CTGTCGCTTCCCATTCTCC			
fhfabp7	Amplification for cloning	F-fhFABP7-PstI	CGGCTGCAGTCATGTCAAAGCTTATCGGTG	72/ Phusion DNA polymerase	15	35
		R-fhFABP7-NotI	ATCGCGGCCGCACTGACAACAGATTGCGCAG			
	Site-directed mutagenesis (N101Q)	FhFABP7_mut_F	TCATGGATTCCAGGAAACCAG	62.6/ Phusion DNA polymerase	90	35
		FhFABP7_mut_R	CGCTGAATTTGGGTCAACTG			

Table S2. Primer sequences used for Real-Time PCR

For-FABP1-RT	CATCACTGAAGTGGTTCGTGAAGTTG
Rev-FABP1-RT	TTTCACATCCCCGACAGTCCACG
For-FABP2-RT	CAAAGACGAGACTTTTGACAACAGA
Rev-FABP2-RT	CTTCACGTCCCCAACAGTAATTG
For-FABP3-RT	CAAAAGGAAACTGATCACAAGTTCC
Rev-FABP3-RT	GGCGGTTTCCTCTTTGAATTCCT
For-FABP4-RT	GGAAGCCTTACTCTCAAACGTG
Rev-FABP4-RT	TTCCATCAGCGGATACTGTCTG
For-FABP5-RT	GCTGAAAATACTAAACCCACTG
Rev-FABP5-RT	TCTGTCTTCATGGTCAGTCC
For-FABP6-RT	AAACACTTCTCCTACACTGTTCTG
Rev-FABP6-RT	TCCGCGGATAGGATTTCAAATTC
For-FABP7-RT	CCATCAAGAGATAGTGGACCA
Rev-FABP7-RT	TCCGTCGTGCAGGTAAACC

Table S3. Specificity matrix of FhFABP primer pairs tested against recombinant plasmids encoding each isoform (Ct and Tm values)

Primers \ Plasmids	FhFABP1	FhFABP2	FhFABP3	FhFABP4	FhFABP5	FhFABP6	FhFABP7
**For/Rev-FABP1-RT**	20.240/80.63	Undetermined	Undetermined	Undetermined	Undetermined	Undetermined	Undetermined
**For/Rev-FABP2-RT**	Undetermined	23.460/81.7	Undetermined	Undetermined	33.772/81.7	Undetermined	Undetermined
**For/Rev-FABP3-RT**	Undetermined	Undetermined	21.471/79.47	Undetermined	34.694/79.37	Undetermined	Undetermined
**For/Rev-FABP4-RT**	Undetermined	Undetermined	Undetermined	21.840/77.43	33.341/77.33	Undetermined	Undetermined
**For/Rev-FABP5-RT**	Undetermined	Undetermined	Undetermined	Undetermined	20.161/76.75	Undetermined	Undetermined
**For/Rev-FABP6-RT**	Undetermined	Undetermined	Undetermined	Undetermined	Undetermined	21.446/75.65	Undetermined
**For/Rev-FABP7-RT**	Undetermined	Undetermined	Undetermined	Undetermined	Undetermined	Undetermined	23.273/76.36

The For/Rev-FABP5-RT primer pair efficiently amplified its target plasmid (Ct = 20.161) and showed only weak amplification of a non-target plasmid, with melting temperatures (Tm) distinct from that of the target amplicon. All primer pairs showed no cross-reactivity when tested with cDNA from all *Fasciola* developmental stages, displaying only a single specific peak corresponding to the target amplicon.

Table S4. FACS antibodies and ELISA kits

Target	Type	Source	Identifier
Human			
TNF	ELISA kit	R&D Systems	DY210
IL-6	ELISA kit	R&D Systems	DY206
IL-10	ELISA kit	R&D Systems	DY217B
CXCL11	ELISA kit	R&D Systems	DY672
TSP-1	ELISA kit	R&D Systems	DY3074
Aqua	Cell death marker	Invitrogen	L34966
CD40	Antibody	BD Pharmingen	555591
CD80	Antibody	BD Horizon	560444
CD83	Antibody	eBioscience	12-0839-42
CD86	Antibody	BD Pharmingen	561129
HLA-DR	Antibody	eBioscience	47-9956-42
CD163	Antibody	BioLegend	333608
CD274 (PD-L1)	Antibody	eBioscience	12-5983-42
CD103	Antibody	BioLegend	350212
CD85k (ILT3)	Antibody	BioLegend	333016

Table S5. Predicted molecular mass and isoelectric point (pI) of *F. hepatica* FABP isoforms.

	Length of the amino acid	Molecular weight (kda)	Izoelectric point
FhFABP1	132	14,712	5,91
FhFABP2	132	14,936	5,93
FhFABP3	132	14,623	9,01
FhFABP4	132	15,073	5,71
FhFABP5	134	15,372	7,96
FhFABP6	161	18,443	4,72
FhFABP7	166	18,742	5,04

Table S6. Prediction of ligand binding for *F. hepatica* FABPs

Protein	Probability	Frequency	Ligand ID	Ligand name
FhFABP1	0.91	23	RTL	Retinol
FhFABP1	0.91	16	RET	Retinal
FhFABP1	0.91	11	REA	Retinoic acid
FhFABP1	0.91	9	PLM	Palmitic acid
FABP1	0.91	7	OLA	Oleic acid
FhABP1	0.91	6	8KS	(1s,2s,3s,4s)-3-{[(naphthalen-1-yl)oxy]carbonyl}-2,4-diphenylcyclobutane-1-carboxylic acid
FhFABP1	0.91	6	HVD	(1'r,2'r)-5'-methyl-6-pentyl-2'-(prop-1-en-2-yl)-1',2',3',4'-tetrahydro[1,1'-biphenyl]-2,4-diol
FhFABP1	0.91	5	RE9	6-(2,3,4,5,6,7-hexahydro-2,4,4-trimethyl-1-metyleneinden-2-yl)-3-methylhexa-2,4-dienoic acid
FhFABP1	0.91	5	ACD	Arachidonic acid
FhFABP1	0.91	5	NC0	2-[(2,3-dihydro-1h-indole-1-carbonyl)amino]benzoic acid
FhFABP3	0.82	29	RET	Retinal
FhFABP3	0.82	25	PLM	Palmitic acid
FhFABP3	0.82	24	RTL	Retinol
FhFABP3	0.82	15	REA	Retinoic acid

FhFABP3	0.82	14	OLA	Oleic acid
FhFABP3	0.82	13	EIC	Linoleic acid
FhFABP3	0.82	9	VCA	Vaccenic acid
FhFABP3	0.82	8	2AN	ANS
FhFABP3	0.82	8	8KS	(1s,2s,3s,4s)-3-{[(naphthalen-1-yl)oxy]carbonyl}-2,4 -diphenylcyclobutane-1-carboxylic acid
FhFABP3	0.82	8	ACD	Arachidonic acid
FhFABP4	0.99	15	REA	Retinoic acid
FhFABP4	0.99	15	OLA	Oleic acid
FhFABP4	0.99	12	PLM	Palmitic acid
FhFABP4	0.99	9	EIC	Linoleic acid
FhFABP4	0.99	8	8KS	(1s,2s,3s,4s)-3-{[(naphthalen-1-yl)oxy]carbonyl}-2,4 -diphenylcyclobutane-1-carboxylic acid
FhFABP4	0.99	7	RET	Retinal
FhFABP4	0.99	7	VCA	Vaccenic acid
FhFABP4	0.99	5	LMC	(2e,4e,6e)-3-methyl-6-(1,3,3-trimethyl-1,3-dihydro-2h -indol-2-ylidene)hexa-2,4-dienal
FhFABP4	0.99	5	HVD	(1'r,2'r)-5'-methyl-6-pentyl-2'-(prop-1-en-2-yl)-1',2',3', 4'-tetrahydro[1,1'-biphenyl]-2,4-diol

FhFABP4	0.99	5	CHD	Cholic acid
FhFABP5	0.77	33	RET	Retinal
FhFABP5	0.77	25	RTL	Retinol
FhFABP5	0.77	16	CHD	Cholic acid
FhFABP5	0.77	13	EIC	Linoleic acid
FhFABP5	0.77	13	PLM	Palmitic acid
FhFABP5	0.77	9	OLA	Oleic acid
FhFABP5	0.77	7	2AN	ANS
FhFABP5	0.77	7	5M8	6-chloranyl-2-methyl-4-phenyl-quinoline-3 -carboxylicacid
FhFABP5	0.77	6	TDZ	Troglitazone
FhFABP5	0.77	6	8KS	(1s,2s,3s,4s)-3-{[(naphthalen-1-yl)oxy]carbonyl}-2,4 -diphenylcyclobutane-1-carboxylic acid
FhFABP6	0.63	26	RET	Retinal
FhFABP6	0.63	19	RTL	Retinol
FhFABP6	0.63	15	CHD	Cholic acid
FhFABP6	0.63	13	EIC	Linoleic acid
FhFABP6	0.63	13	OLA	Oleic acid
FhFABP6	0.63	13	PLM	Palmitic acid

FhFABP6	0.63	10	5M8	6-chloranyl-2-methyl-4-phenyl-quinoline-3 -carboxylicacid
FhFABP6	0.63	7	2AN	ANS
FhFABP6	0.63	6	TDZ	Troglitazone
FhFABP6	0.63	6	VCA	Vaccenic acid
FhFABP7	0.65	25	RET	Retinal
FhFABP7	0.65	12	BLR	Bilirubin
FhFABP7	0.65	10	LSR	1,3,3-trimethyl-2-[(1e,3e)-3-methylpenta-1,3 -dien-1-yl]cyclohexene
FhFABP7	0.65	9	OLA	Oleic acid
FhFABP7	0.65	5	T4B	((2'-(5-ethyl-3,4-diphenyl-1h-pyrazol-1-yl)-3 -biphenylyl)oxy)acetic acid
FhFABP7	0.65	5	EIC	Linoleic acid
FhFABP7	0.65	5	REA	Retinoic acid
FhFABP7	0.65	4	NC0	2-[(2,3-dihydro-1h-indole-1-carbonyl)amino] benzoic acid
FhFABP7	0.65	3	W7Z	(8s)-5-[(3-chloro-2-methylphenoxy)methyl]-2 -phenyl[1,2,4]triazolo[1,5-a]pyrimidin-7(4h)-one
FhFABP7	0.65	3	RH6	(2e)-3-[7-(diethylamino)-2-oxo-2h-1-benzopyran-3 -yl]prop-2-enal, bound form

FhFABP7	0.65	2	JLO	(2e,4e)-3-methyl-5-(2,3,6,7-tetrahydro-1h,5h-pyrido [3,2,1-ij]quinolin-9-yl)penta-2,4-dienal

Supplementary figures

Figure S1.

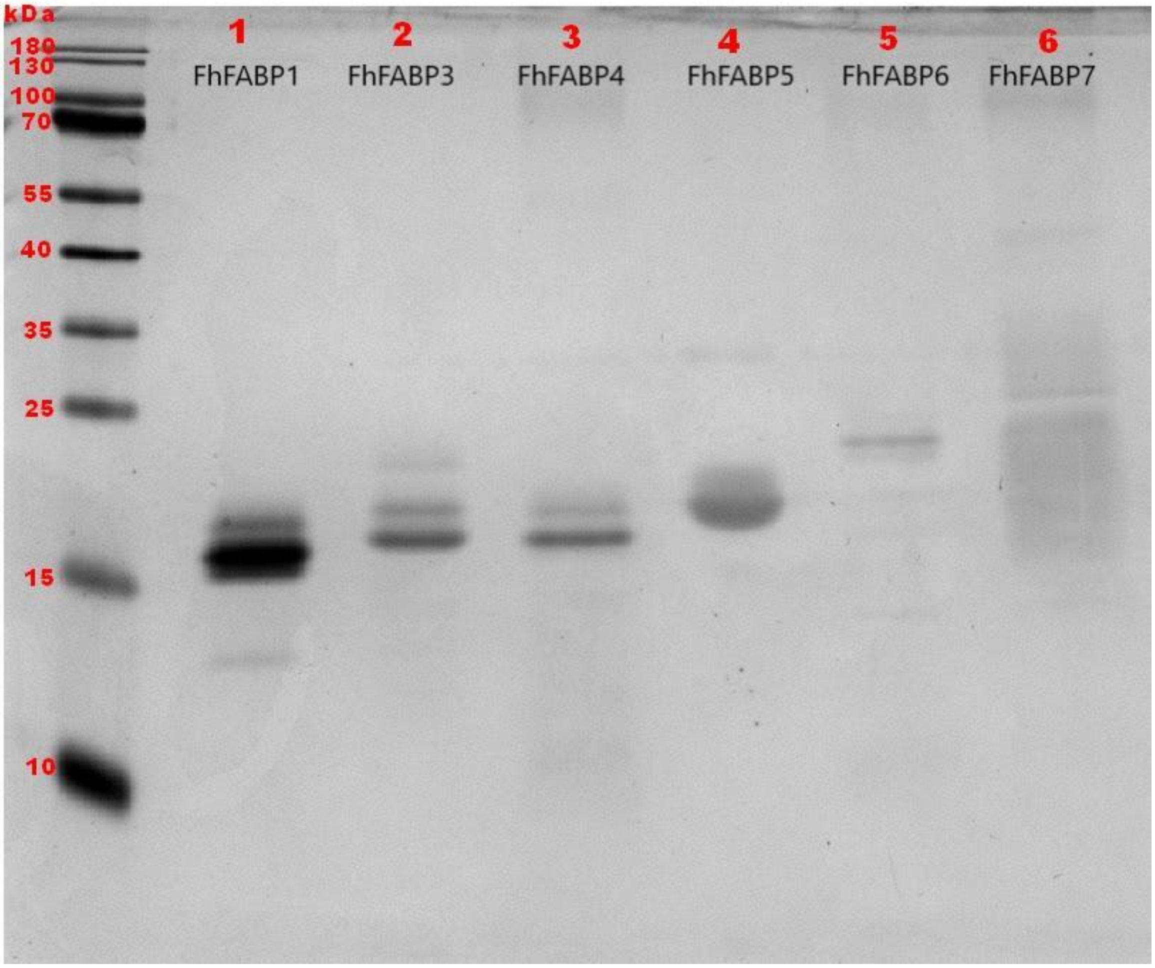

Fig S1. Expression of *F. hepatica* FABPs. Lane (M) contains molecular weight markers; lanes (1) through (6) contain purified recombinant proteins: (1) FhFABP1, (2) FhFABP3, (3) FhFABP4, (4) FhFABP5, (5) FhFABP6, and (6) FhFABP7. The molecular masses (kDa) are indicated on the left. The gel was silver-stained.

Figure S2.

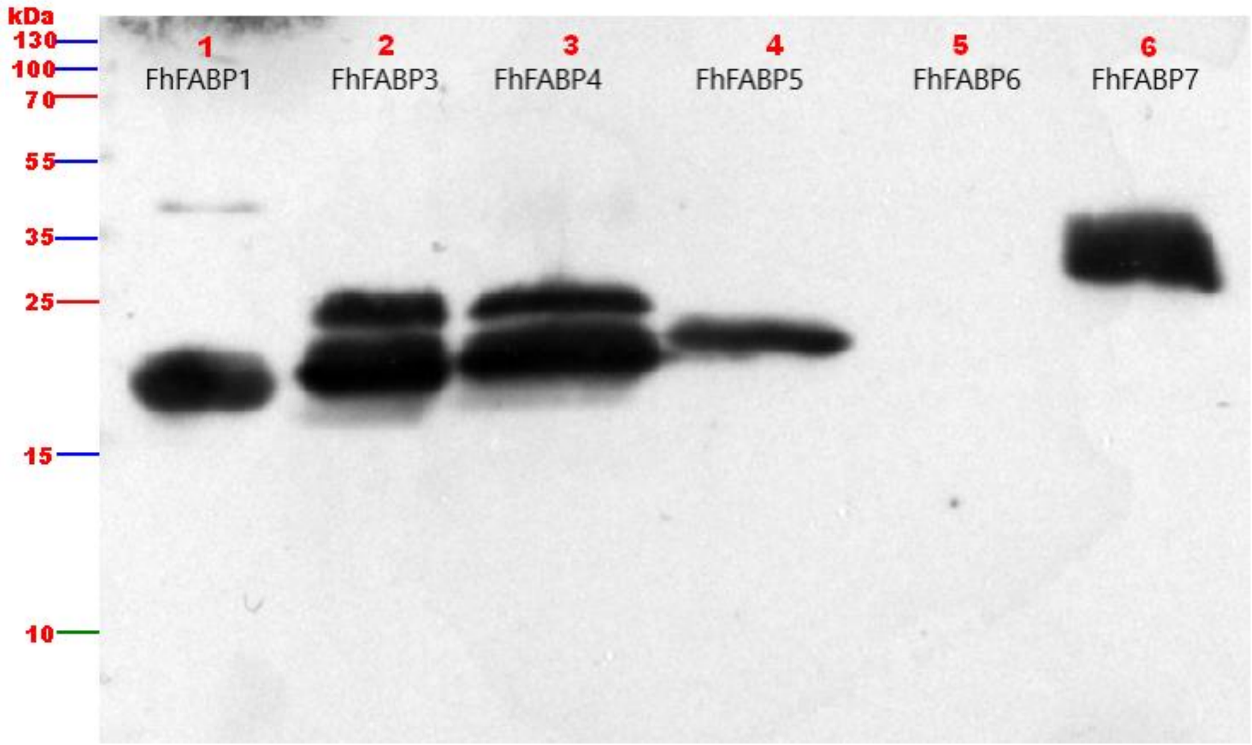

Fig S2. Western blot analysis showing the specificity of rat anti-FABP1 serum to recombinant *F. hepatica* FABP isoforms. Lane (1) contains purified recombinant FhFABP1; lanes (2) through (6) contain FhFABP3, FhFABP4, FhFABP5, FhFABP6, and FhFABP7, respectively. The molecular masses (kDa) are indicated on the left. Uncropped blot below.

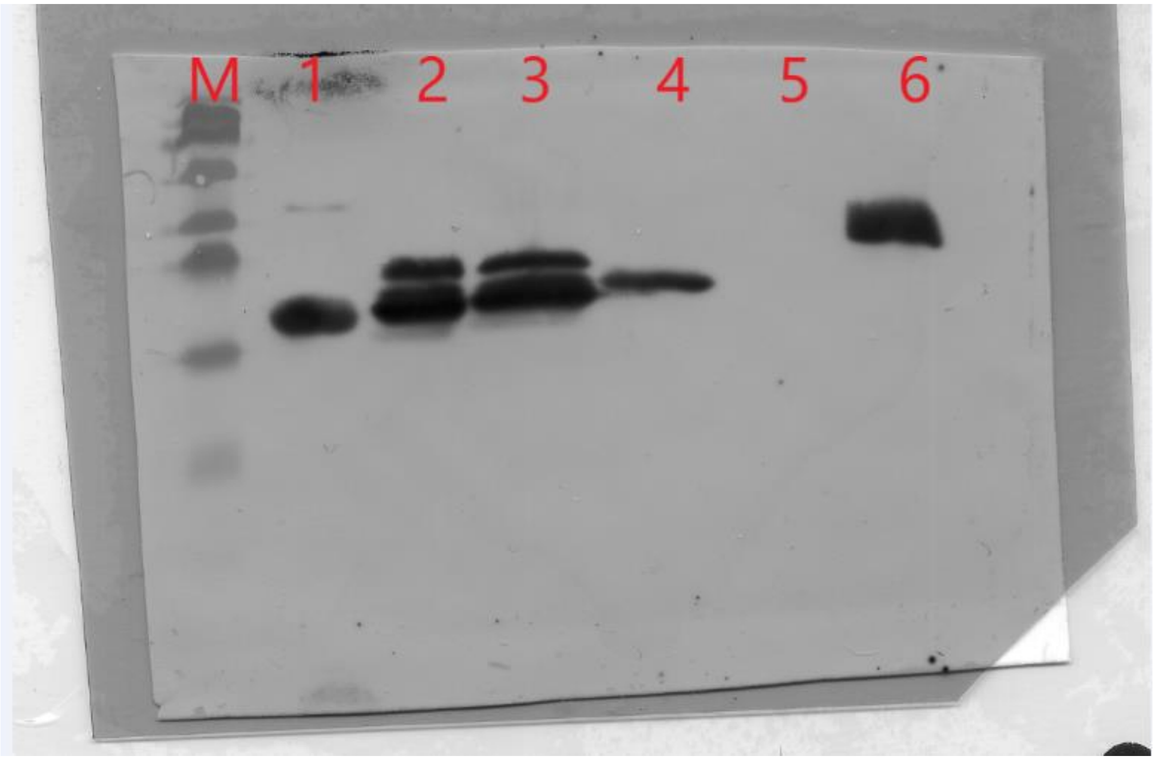
M
1
2
3
4
5
6

Figure S3.

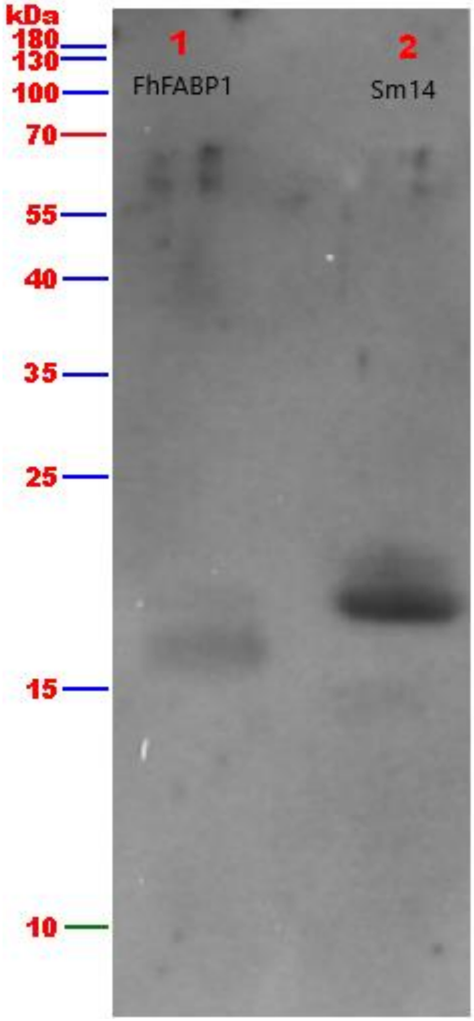

Fig S3. Western blot identification of *Schistosoma mansoni* Sm14 using rat anti-FhFABP1 serum. Lane (1) contains FhFABP1 (positive control), and lane (2) contains Sm14. Uncropped blot below.

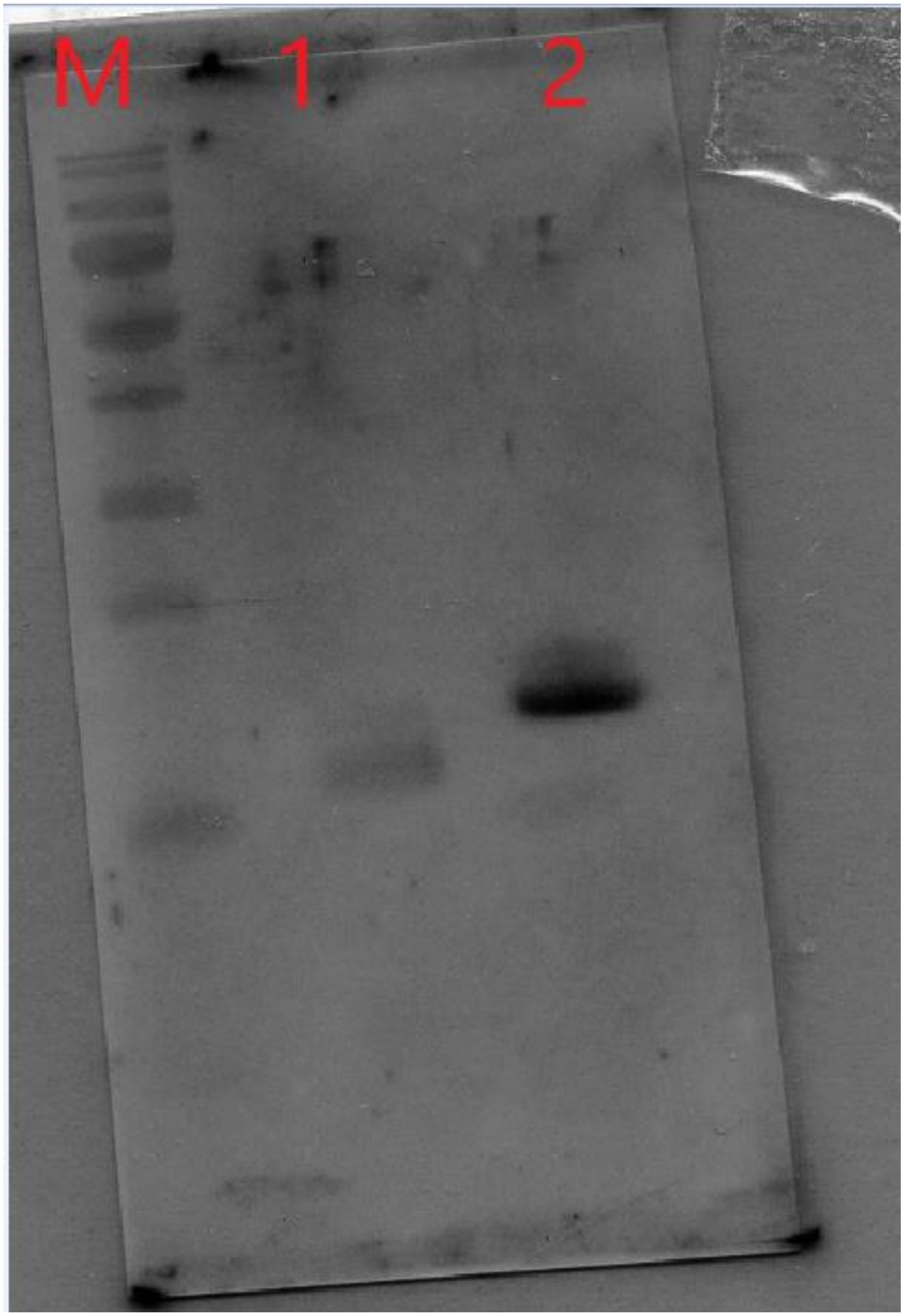
M
1
2

Figure S4.

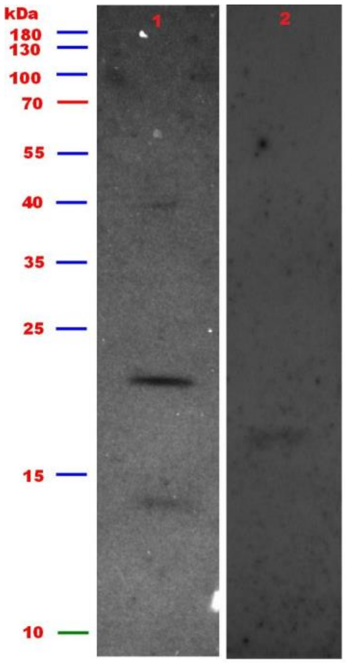

Fig S4. Identification of *F. hepatica* proteins using rat anti-FABP1 serum in Western blot analysis. Lane (1) contains homogenate from adult flukes, and lane (2) contains excretory-secretory (ES) products from adult flukes. Uncropped blots below.

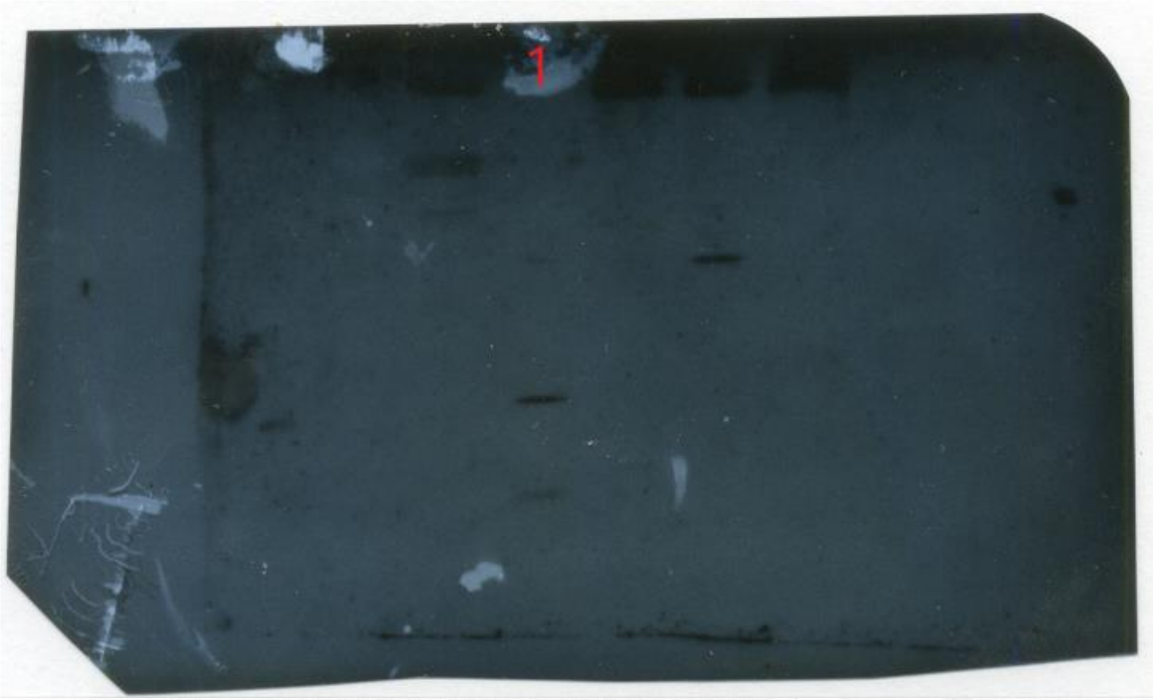
1

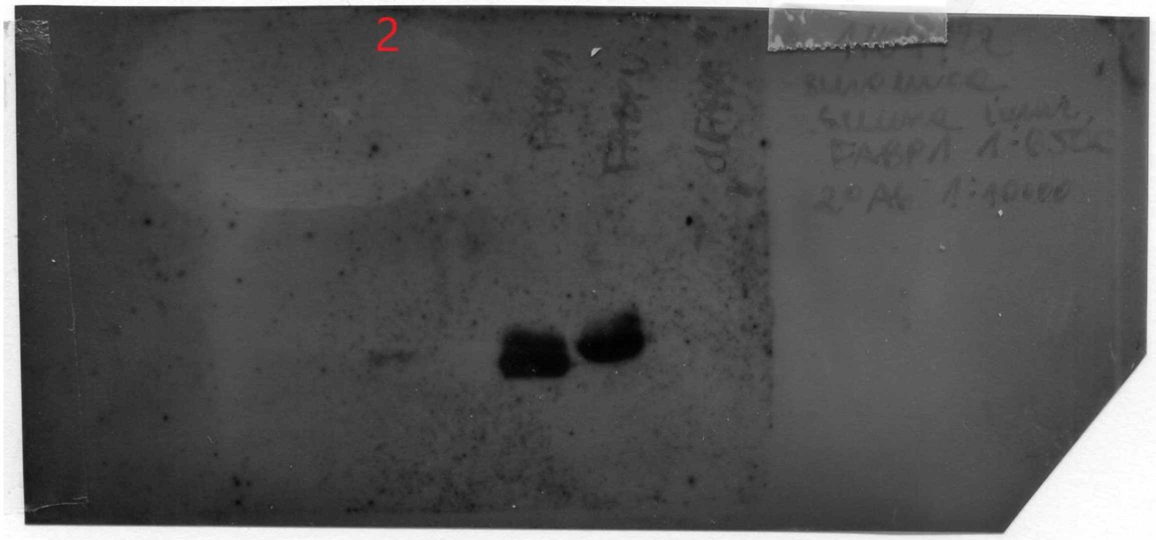
2
